# The benign nature and rare occurrence of cardiac myxoma as a possible consequence of the limited cardiac proliferative/ regenerative potential: a systematic review

**DOI:** 10.1186/s12885-023-11723-3

**Published:** 2023-12-18

**Authors:** Ovais Shafi, Ghazia Siddiqui, Hassam A. Jaffry

**Affiliations:** Sindh Medical College - Jinnah Sindh Medical University / Dow University of Health Sciences, Karachi, Pakistan

**Keywords:** Cardiac myxoma, Cardiogenesis, Cardiomyocytes, Cardiac regeneration, Tumorigenesis, Stem cells, Tumor suppressors, Cardiac progenitor cells

## Abstract

**Background:**

Cardiac Myxoma is a primary tumor of heart. Its origins, rarity of the occurrence of primary cardiac tumors and how it may be related to limited cardiac regenerative potential, are not yet entirely known. This study investigates the key cardiac genes/ transcription factors (TFs) and signaling pathways to understand these important questions.

**Methods:**

Databases including PubMed, MEDLINE, and Google Scholar were searched for published articles without any date restrictions, involving cardiac myxoma, cardiac genes/TFs/signaling pathways and their roles in cardiogenesis, proliferation, differentiation, key interactions and tumorigenesis, with focus on cardiomyocytes.

**Results:**

The cardiac genetic landscape is governed by a very tight control between proliferation and differentiation-related genes/TFs/pathways. Cardiac myxoma originates possibly as a consequence of dysregulations in the gene expression of differentiation regulators including Tbx5, GATA4, HAND1/2, MYOCD, HOPX, BMPs. Such dysregulations switch the expression of cardiomyocytes into progenitor-like state in cardiac myxoma development by dysregulating Isl1, Baf60 complex, Wnt, FGF, Notch, Mef2c and others.

The Nkx2–5 and MSX2 contribute predominantly to both proliferation and differentiation of Cardiac Progenitor Cells (CPCs), may possibly serve roles based on the microenvironment and the direction of cell circuitry in cardiac tumorigenesis. The Nkx2–5 in cardiac myxoma may serve to limit progression of tumorigenesis as it has massive control over the proliferation of CPCs. The cardiac cell type-specific genetic programming plays governing role in controlling the tumorigenesis and regenerative potential.

**Conclusion:**

The cardiomyocytes have very limited proliferative and regenerative potential. They survive for long periods of time and tightly maintain the gene expression of differentiation genes such as Tbx5, GATA4 that interact with tumor suppressors (TS) and exert TS like effect. The total effect such gene expression exerts is responsible for the rare occurrence and benign nature of primary cardiac tumors. This prevents the progression of tumorigenesis. But this also limits the regenerative and proliferative potential of cardiomyocytes. Cardiac Myxoma develops as a consequence of dysregulations in these key genes which revert the cells towards progenitor-like state, hallmark of CM. The CM development in carney complex also signifies the role of TS in cardiac cells.

## Background

Primary tumors of the heart are exceedingly rare, with nearly 90% of them classified as benign [1]. This study focuses on a particular subtype of benign cardiac tumors known as cardiac myxomas (CM), primarily because they are the most prevalent among primary benign cardiac tumors [1]. Cardiac myxomas, despite their benign nature, hold significant relevance in cardiac research and clinical practice. CM are benign tumors of the heart. They are multipotent with mesenchymal stem cell-like nature. To comprehend the significance of CM and their potential implications for cardiomyocyte biology and cardiac regeneration, it is crucial to first understand the general concepts surrounding cardiac regeneration. The adult human heart has limited regenerative capacity, a characteristic that poses significant challenges in addressing cardiac diseases [2]. This limited regenerative potential of cardiomyocytes has sparked interest in understanding the mechanisms governing cardiac cell fate and the factors that might influence their regenerative and proliferative abilities [3].

Histopathological features of CM often reveal areas of hypercellularity, necrosis, and atypia [1, 3, 4]. It is within these intricate patterns that the transformation of cardiomyocytes into cardiac progenitor-like cells, a distinctive hallmark of cardiac myxoma, becomes evident [5, 6]. These areas of hypercellularity suggest the presence of cells in various stages of differentiation, reminiscent of cardiac progenitors [7, 8]. Furthermore, the presence of atypia hints at the dynamic reprogramming of cardiac cells, as they shift from their terminally differentiated state toward a more primitive, progenitor-like phenotype. The histopathological landscape of CM serves as a visual representation of the intriguing process by which cardiomyocytes seem to undergo transformation into progenitor-like cells, hallmark of CM [9, 10].

### Current challenges and gaps

The precise etiology of CM remains elusive, and despite some studies hinting at the possible role of transcription factor Nkx2–5 in CM development, our understanding of CM’s origin and the factors involved is far from comprehensive [11–15].

The possible role of limited regenerative and proliferative potential of cardiomyocytes in the development of primary cardiac tumors such as CM is not yet fully understood [4, 5], and how this is related to the benign nature of CM [6, 8].

### Objectives of the study

CM are benign tumors of the heart. They are multipotent with mesenchymal stem cell-like nature [9, 10, 16–18]. This study intends to contribute to a deeper understanding of cardiomyocyte biology and the factors that possibly influence their resistance to neoplastic transformations [19]. It investigates how the resistance to malignant transformation of CM may possibly be related to the limited proliferative and regenerative potential of cardiomyocytes [7].

Our aim is to explore the relationship between CM and the limited regenerative potential of cardiomyocytes. By investigating key cardiac transcription factors, genes, signaling pathways, and other mechanisms, we seek to shed light on the development of CM and its potential implications for cardiac regeneration [20–24].

## Methods

### Article screening and inclusion

The process of article screening and inclusion was meticulously conducted to ensure that only relevant and high-quality literature was incorporated into this study. The aim was to identify and evaluate articles that provided insight into the roles of cardiac genes, transcription factors (TFs), and signaling pathways in cardiogenesis, cardiomyocyte development, proliferation, differentiation, tumorigenesis, and their connection to Cardiac Myxoma (CM). The literature search and data collection commenced in January 2019 and concluded in February 2022. During the revision process, additional literature searches were conducted and referenced up to October 2022. The study’s timeline facilitated a significant focus on genes, TFs, and pathways that are fundamental to the regulation of cardiac development, lineage commitment, and the maintenance of cardiac identity. The extensive duration enabled a thorough search across various databases and resources, ensuring that no relevant information was overlooked. Understanding the roles of these pivotal cardiac genes and TFs is crucial for unraveling the complexities of CM development. Their participation in balancing proliferation and differentiation processes is particularly intriguing and necessitated a wide search horizon to encompass the evolving body of knowledge in this field. This endeavor involved a comprehensive exploration of key cardiac genes, transcription factors (TFs), and signaling pathways, each of which plays multifaceted roles in cardiogenesis, cardiomyocyte development, proliferation, differentiation, tumor suppression or tumorigenesis.

1. **Database selection and search strategy:** The search process commenced with a thorough selection of databases renowned for their comprehensive coverage in the field of biomedical research. Three primary databases were: PubMed, MEDLINE, and Google Scholar. These platforms were selected due to their accessibility and comprehensive indexing, allowing us to obtain a broad spectrum of literature.
2. **Multi-step screening process:** A multi-step screening process was systematically implemented to identify articles aligning with our predefined inclusion and exclusion criteria. This process aimed at meticulously filtering through a large pool of potentially relevant studies to ensure that only the most pertinent articles were considered for inclusion in our study.

### Title and abstract screening

Initially, articles were screened based on their titles and abstracts. This preliminary step served as an effective means of identifying articles that exhibited direct relevance to the research objectives of this study. Articles that clearly did not pertain to cardiac genes, TFs, signaling pathways, or cardiomyocyte biology were eliminated.

### Full-text review

Articles that passed the title and abstract screening phase underwent a comprehensive full-text review. During this in-depth assessment, we scrutinized each article to determine its relevance to the study’s focus areas. Those that did not offer valuable insights into the roles of cardiac genes, TFs, and signaling pathways in the context of cardiogenesis, cardiomyocyte development, proliferation, differentiation, tumorigenesis, or CM were excluded.

3. **Data extraction:** Following the final selection of articles, relevant data was extracted from each article, encompassing key findings and outcomes that were significant to our study objectives. This process aimed to ensure that the data extracted was relevant and provided valuable insights into the complex interplay of cardiac genes, TFs, and signaling pathways.

#### Inclusion criteria, selection of genes, transcription factors, and signaling pathways

To be deemed eligible for inclusion in our study, articles had to be directly related to key cardiac transcription factors/genes and signaling pathways involved in cardiogenesis, with a specific emphasis on the developmental biology of cardiomyocytes and cardiomyocyte differentiation. Articles that did not meet these specific criteria were excluded. The choice of specific cardiac genes, TFs, and signaling pathways for investigation was guided by their well-established roles in cardiogenesis, cardiomyocyte development, proliferation, and differentiation. Furthermore, these factors were also examined in the context of tumor suppression, tumorigenesis, and their relevance to CM. While the factors we selected are indeed significant contributors to cardiac biology and pathology, it is essential to acknowledge that other factors may also play a role in the processes under investigation. However, due to the defined scope of our study, these factors were beyond the current study’s scope.

The following genes/TFs and signaling pathways were investigated for their role in cardiogenesis/cardiomyocyte development, proliferation, differentiation, tumor suppression, tumorigenesis and in CM: *Isl1, Brg1/Baf60 – Smarcd3 complex, Nkx2–5, GATA4, Tbx5, Mef2c, HAND1/2, MYOCD, MSX2, HOPX, Wnt-signaling pathway, Notch, FGF, BMPs.*

These key cardiac genes/TFs play pivotal roles in cardiac development by guiding lineage commitment, balancing proliferation and differentiation, regulating essential processes, and maintaining cardiac identity.

This study adheres to relevant PRISMA guidelines (Preferred Reporting Items for Systematic Reviews and Meta-Analyses).

### Rationale for screening and inclusion

It is deeply rooted in the pivotal significance of certain cardiac genes, transcription factors (TFs), and signaling pathways in unraveling the complex process of cardiac tumorigenesis, with a primary focus on cardiac myxomas (CM). The selection of these specific factors is driven by their unique and critical roles, which have the potential to shed light on CM development. These include Isl1, a key controller of cardiomyocyte cell fate, highly expressed in multipotent cardiac progenitor cells (CPCs), which not only specifies cardiac lineage and differentiation but also exhibits interactions with Nkx2–5 and Estrogen Receptor Alpha, suggesting its potential involvement in CM. The Brg1/Baf60 – Smarcd3 Complex acts as a crucial transcriptional regulator, inducing CPC proliferation, and its defects are linked to differentiation anomalies, potentially collaborating with the Wnt signaling pathway in promoting tumorigenesis. Nkx2–5, among the earliest cardiac-specific patterning genes, plays a central role in inducing cardiac programming in CPCs, enhancing differentiation, and interacting with Tbx5 and GATA4, especially with its upregulated expression in CM, possibly contributing to CM heterogeneity. GATA4, a master regulator of genes pivotal for cardiogenesis, significantly influences cardiac morphogenesis, survival, and differentiation, with decreased expression leading to cardiomyocyte reversion to a progenitor-like state. Inclusion of Tbx5 is essential due to its ability to boost the expression of other cardiogenic TFs, thereby guiding CPC differentiation while suppressing non-cardiac gene expression and potentially contributing to CM heterogeneity. The Mef2c gene is incorporated as it contributes to CPC proliferation and forms complexes with key cardiac TFs, participating in cardiac morphogenesis and enhancing differentiation, potentially collaborating with the Wnt pathway and Isl1 in generating CPC-like states. The HAND1/2, with regulatory roles in cardiogenesis, including the enhancement of both proliferation with Nkx2–5 and differentiation with GATA4, is of particular interest as it may act as a tumor suppressor and possibly becomes downregulated in CM development, making it crucial to understand the multifaceted roles of these TFs in both normal cardiac development and CM tumorigenesis. The MYOCD gene is another crucial factor under investigation, as it regulates CPC growth arrest and governs CPC stemness. Understanding the role of MYOCD can offer insights into the factors that maintain the unique characteristics of CM. MSX2, included for its interactions with HAND1/2 in regulating gene expression and its role in enhancing CPC proliferation, is of particular interest due to its potential involvement in promoting progenitor-like states in advanced CM. HOPX, expressed as CPCs commit to the cardiomyocyte fate, is essential for enhancing cardiomyocyte differentiation and acting as a tumor suppressor. The investigation of HOPX provides insights into how its downregulation may contribute to CM development. The inclusion of the Wnt Signaling Pathway is vital as it is involved in CPC renewal and maintenance, processes that are essential in understanding the regenerative potential of cardiac cells. Its role in enhancing CPC stemness and contributing to cardiomyocyte dedifferentiation in CM development is central to this research. The FGF Signaling Pathway is included as it drives stem cell differentiation into CPCs and forms complexes that regulate differentiation and proliferation. The potential for its dysregulation to lead to the reversion of cardiomyocytes toward progenitor-like states in CM development highlights its significance in this study. BMPs, through their role in downregulating progenitor genes in CPCs and enhancing cardiomyocyte differentiation, are vital in understanding the control of cardiac cell fate and its impact on CM development. The Notch Signaling Pathway, participating in cardiac morphogenesis and regulating cardiomyocyte proliferation and differentiation, is included to uncover its potential collaboration with Isl1 and Mef2c in CM development. The comprehensive exploration of these key genes, TFs, and signaling pathways is essential to provide valuable insights into their roles in cardiogenesis, proliferation, differentiation, and their potential contributions to the development of cardiac myxomas.

#### Assessment of article quality and potential biases

During the article screening and inclusion process, the quality of the selected articles and the assessment of potential biases were pivotal aspects to ensure the rigor and reliability of the research findings.

1. **Quality assessment:** The first step in quality assessment involved evaluating the methodological rigor of the selected articles. This entailed a careful examination of the study design, data collection methods, and analyses conducted in those studies. The quality of evidence was considered when determining the significance of the study’s findings. Articles that demonstrated sound methodology, such as well-designed studies/experiments, controlled variables, and appropriate scientifically sound data, were considered of higher quality. The fact that the selected articles had undergone a peer-review process was also a significant indicator of quality. Peer-reviewed articles are subject to scrutiny by experts in the field, ensuring the validity and credibility of the research. The quality of evidence presented in the selected articles was also a focus of the assessment. High-quality evidence often comes from properly conducted reviews or well-designed studies with rigorous data collection methods and robust analyses.
2. **Potential biases assessment:**

Publication bias: The potential for publication bias was addressed. This bias can occur when only studies with positive or significant results are published, leading to an overestimation of effects. To minimize this bias, studies that provided a balanced representation of both positive and negative results were actively sought. A comprehensive search strategy, including databases like Google Scholar, was adopted to include a wide range of published articles.

Selection bias: To assess selection bias, predefined and transparent inclusion criteria was applied to minimize subjectivity. Articles were selected based on their relevance to this study’s objectives, and this process adhered to predefined criteria. This approach reduced the risk of subjectivity in article selection.

Reporting bias: Reporting bias occurs when studies selectively report certain outcomes while omitting others. Articles were checked for inconsistencies or missing data to make sure that such studies do not mislead the findings of this study. To identify and address reporting bias, multiple detailed reviews of the methodologies and results were conducted for all the selected articles.

By ensuring that high-quality, peer-reviewed studies were included and potential biases were assessed, this study aimed to provide a robust foundation for results and conclusions presented in the study. This enhanced the reliability and credibility of the study, making it a valuable contribution to the field of cardiac genetics and its role in tumorigenesis.

### Language and publication restrictions

We restricted our selection to publications in the English language. There were no limitations imposed on the date of publication. Unpublished studies were not included in our analysis.

## Results

**Fig. 1:**
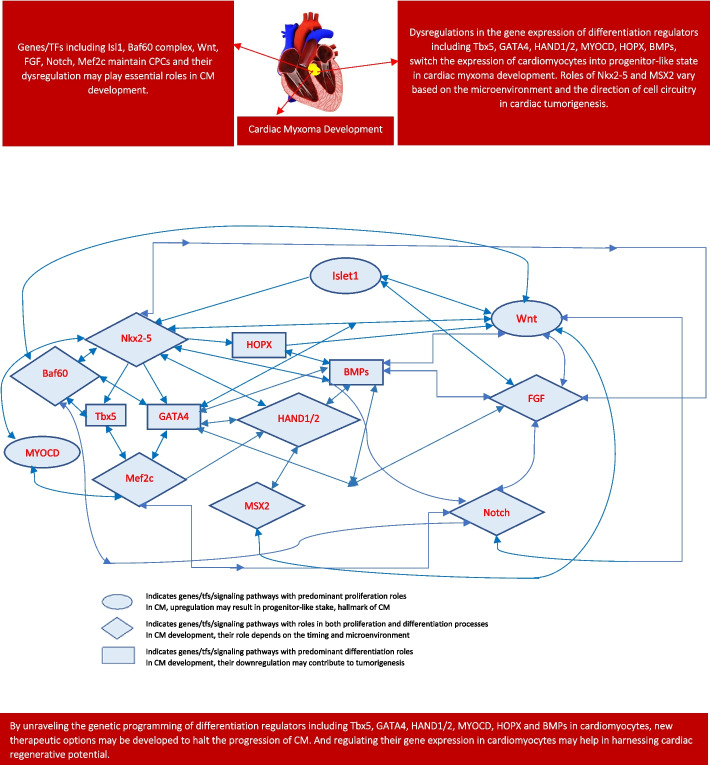
Landscape of genes/tfs/signaling pathways involved in cardiac development and their possible roles in CM

**Fig. 2:**
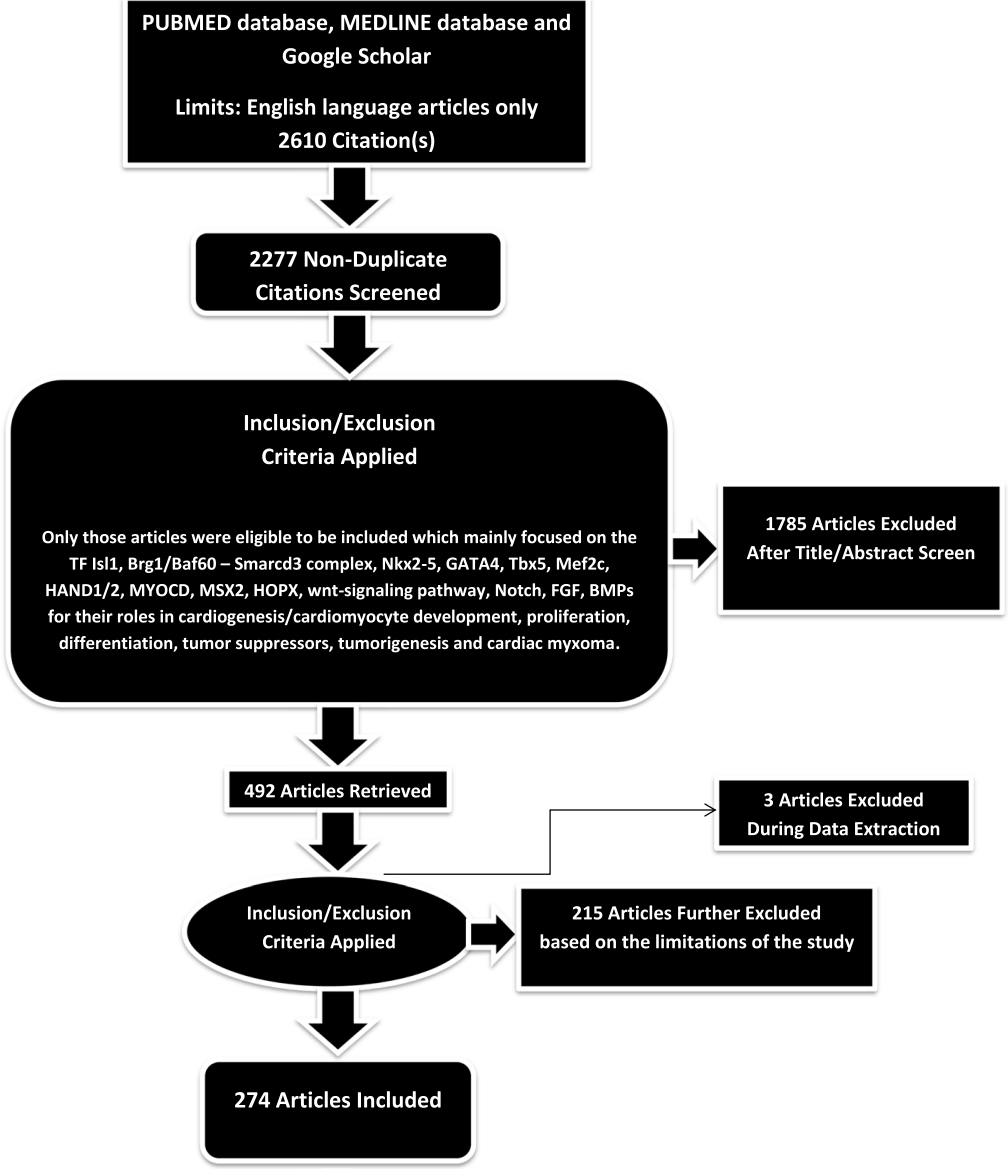
PRISMA flow diagram: This figure only highlights the methodology of the study in relation to its limitations. This figure represents graphically the flow of citations in the study

A total of 2610 articles were identified using database searching, and 2277 were recorded after duplicates removal. One thousand seven hundred eighty-five (1785) were excluded after screening of title/abstract, 215 articles were further excluded from consideration based on a more detailed review of the full texts. These exclusions were primarily due to factors such as non-conformity with the study focus, insufficient methodological rigor, or data that did not align with our research questions. and 3 articles were excluded during data extraction. Finally, 274 articles were included (79 were review articles, 1 was clinical trial, 105 were in vivo studies and 89 were in vitro studies). Ultimately, 2 duplicate references were also deleted during the final checks. Figure 1 illustrates the landscape of genes/transcription factors/signaling pathways involved in cardiac development and their potential roles in Cardiac Myxoma. The flow of citations is represented in Fig. 2.

### Significance of cardiac genes/TFs in investigating cardiac tumorigenesis: an overview of the study

1. Isl1: Controls cardiomyocyte cell fate, strongly expressed in multipotent cardiac progenitor cells (CPCs). Specifies cardiac lineage and differentiation. Interactions with Nkx2–5 and Estrogen Receptor Alpha suggest potential involvement.
2. Brg1/Baf60 – Smarcd3 Complex: Acts as a transcriptional regulator. Induces CPC proliferation; defects in this complex lead to differentiation defects. May collaborate with the Wnt signaling pathway to promote tumorigenesis.
3. Nkx2–5: Among the earliest cardiac-specific patterning genes, induces cardiac programming in CPCs. Enhances differentiation; interacts with Tbx5 and GATA4. Upregulated expression in CM; potential contribution to CM heterogeneity.
4. GATA4: Regulates genes crucial for cardiogenesis. Influences morphogenesis, survival, and differentiation. Reduced expression leads to cardiomyocyte reversion to a progenitor-like state.
5. Tbx5: Boosts expression of other cardiogenic TFs. Enhances CPC differentiation; suppresses non-cardiac gene expression. May contribute to CM heterogeneity.
6. Mef2c: Contributes to CPC proliferation; forms complexes with key cardiac TFs. Involved in cardiac morphogenesis; enhances differentiation. May collaborate with Wnt and Isl1 in generating CPC-like states.
7. HAND1/2: Regulates cardiogenesis; enhances proliferation with Nkx2–5 and differentiation with GATA4. May act as a tumor suppressor; possibly downregulated in CM development.
8. MYOCD: Regulates CPC growth arrest. Governs CPC stemness. May contribute to CM’s benign nature and rarity.
9. MSX2: Interacts with HAND1/2 to regulate gene expression. Enhances CPC proliferation. May promote progenitor-like states in advanced CM.
10. HOPX: Expressed as CPCs commit to cardiomyocyte fate. Enhances cardiomyocyte differentiation; acts as a tumor suppressor. Downregulation may contribute to CM development.
11. Wnt Signaling Pathway: Involved in CPC renewal and maintenance. Enhances CPC stemness; contributes to cardiomyocyte dedifferentiation in CM development.
12. FGF Signaling Pathway: Drives stem cell differentiation into CPCs. Forms complexes regulating differentiation and proliferation; dysregulation may reverse cardiomyocytes toward progenitor-like states in CM development.
13. BMPs: Downregulates progenitor genes in CPCs. Enhances cardiomyocyte differentiation. Downregulation may contribute to CM.
14. Notch Signaling Pathway: Participates in cardiac morphogenesis. Regulates cardiomyocyte proliferation and differentiation. May collaborate with Isl1 and Mef2c in CM development.

Based on the objectives of the study, the comprehensive exploration of key genes, TFs, and signaling pathways is given below. It is capable of offering valuable insights into their roles in cardiogenesis, proliferation, differentiation, and their potential contributions to the development of cardiac myxomas.

Table 1 presents an overview of cardiac genes, transcription factors, and signaling pathways, shedding light on their pivotal roles in cardiogenesis, proliferation/ differentiation, and their possible role in involvement in Cardiac Myxoma (CM) development.

**Table 1:** Cardiac genes/transcription factors/signaling pathways and their roles in cardiogenesis, proliferation/differentiation, and in CM development

Genes/TFs/Signaling pathways	Role in Cardiogenesis	Role in Proliferation/Differentiation	Possible Role in CM Development	Abbreviations
Isl1	Controls cardiomyocyte fate; highly expressed in multipotent CPCs [25–29]	Directs cardiac cell lineage and differentiation [30–39].	Potential involvement in CM development; interacts with Nkx2–5 and Estrogen Receptor Alpha [40–45]	Isl1: Islet-1 CPCs: Cardiac Progenitor Cells CM: Cardiac Myxoma Nkx2–5: NK2 Homeobox 5
Brg1/Baf60 – Smarcd3 complex	Acts as transcriptional regulator [46–48].	Induces proliferation of CPCs. Differentiation becomes defective when there are defects in this complex [49–55].	Baf60 complex may work with Wnt signaling pathway to promote tumorigenesis [47, 48].	Brg1/Baf60 – Smarcd3 complex - Brahma-Related Gene 1/Brg1 Associated Factor 60, SMARCD3 Complex CPCs: Cardiac Progenitor Cells Wnt pathway - Wingless/Integrated Pathway
Nkx2–5	Among the very first cardiac specific patterning genes [56, 57].	Induces cardiac programming in CPCs. Enhances differentiation when interacting with Tbx5 and GATA4 [58–68].	Upregulated expression in CM development. Potential contributor to the heterogeneity that exists in CM [69–72].	Nkx2–5: NK2 Homeobox 5 CPCs: Cardiac Progenitor Cells Tbx5: T-Box 5 GATA4: GATA Binding Protein 4 CM: Cardiac Myxoma
GATA4	Important regulator of genes involved in cardiogenesis [73, 74].	Plays a significant role in morphogenesis, survival and regulates differentiation [75–79].	Decreased GATA4 expression changes cadiomyocytes into progenitor-like state [80, 81].	GATA4: GATA Binding Protein
Tbx5	Increases the expression of other cardiogenic TFs [82–86].	Enhances differentiation of CPCs into cardiomyocytes [87–94].	Suppresses the expression of genes involved in non-cardiac cell types. Potential contributor to the heterogeniety in CM [95, 96].	Tbx5: T-Box 5 CPCs: Cardiac Progenitor Cells CM: Cardiac Myxoma
Mef2c	Contributes to the proliferation of CPCs and forms complexes with key cardiac TFs [97–100].	Involved in cardiac morphogenesis. Works with the GATA4 and Tbx5 to enhance differentiation [101].	In CM development, it may work with Wnt and Isl1 with resulting emergence of CPC-like state [102–105].	Mef2c - Myocyte Enhancer Factor 2C GATA4: GATA Binding Protein 4 Tbx5: T-Box 5 Wnt pathway - Wingless/Integrated Pathway Isl1: Islet-1 CM: Cardiac Myxoma CPCs: Cardiac Progenitor Cells
HAND1/2	Works with key TFs to regulate cardiogenesis [106, 107].	Enhances proliferation with Nkx2–5. On interaction with GATA4, it enhances the differentiation of cardiomyocytes [108, 109].	It also acts as a tumor suppressor and is possibly downregulated in CM development [110]	HAND1/2: Heart- and Neural Crest-Derived Transcript 1/2 Nkx2–5: NK2 Homeobox 5 GATA4: GATA Binding Protein 4 CM: Cardiac Myxoma
MYOCD	Regulates the growth arrest of CPCs in cardiogenesis [111]	Regulates the stemness of CPCs [112, 113].	May contribute to the benign nature and rare occurrence of CM [111].	MYOCD: Myocardin CPCs: Cardiac Progenitor Cells CM: Cardiac Myxoma
MSX2	In cardiogenesis, MSX2 interacts with HAND1/2 and they regulate the gene expression of each other [114, 115]	Enhances the proliferation of CPCs [116]	In advanced CM, it may possibly contribute to the tumorigenesis by promoting progenitor-like state [116].	MSX2: Msh Homeobox 2 HAND1/2: Heart- and Neural Crest-Derived Transcript 1/2 CPCs: Cardiac Progenitor Cells CM: Cardiac Myxoma
HOPX	Expressed when CPCs become committed to cardiomyocyte fate [117].	Enhances differentiation of cardiomyocytes. Also acts as a tumor suppressor [118, 119].	Downregulation of HOPX may contribute to the CM development [117–119].	HOPX: Homeodomain-Only Protein X CPCs: Cardiac Progenitor Cells CM: Cardiac Myxoma
Wnt signaling pathway	Involved in the renewal and maintenance of CPCs [120, 121]	Enhances stemness in the CPCs [122, 123]	Contributes to the dedifferentiation of cardiomyocytes into progenitor-like state in CM development [124, 125]	Wnt: Wingless/Integrated Pathway CPCs: Cardiac Progenitor Cells CM: Cardiac Myxoma
FGF signaling pathway	Plays role in differentiating the pluripotent stem cells into CPCs [126–129]	Forms complexes with the key regulators of cell cycle to govern the differentiation and proliferation processes [130–132].	Dysregulations and decline in the FGF signaling may cause reversal of cardiomyocytes towards progenitor-like state in CM development [133, 134].	FGF: Fibroblast Growth Factor CPCs: Cardiac Progenitor Cells CM: Cardiac Myxoma
BMPs	Downregulate the expression of the progenitor genes in CPCs [135–137].	Enhances the differentiation of cardiomyocytes [138, 139].	The downregulation of BMP expression may contribute to the CM development [136, 137].	BMPs: Bone Morphogenetic Proteins CPCs: Cardiac Progenitor Cells CM: Cardiac Myxoma
Notch signaling pathway	Involved in the process of cardiac morphogenesis [140–143].	Involved in the process of cardiomyocyte proliferation and differentiation [144–146].	May work with Isl1 and Mef2c to contribute to the CM development [140, 141, 144].	Notch: Notch Signaling Pathway Isl1: Islet-1 Mef2c: Myocyte Enhancer Factor 2C CM: Cardiac Myxoma
PRKAR1A	Regulates the c-AMP protein kinase A signaling [147].	Also acts as a tumor suppressor and contributes to the process of differentiation [148, 149].	The mutation in PRKAR1A causes myxomas and carney complex [150, 151].	PRKAR1A: Protein Kinase cAMP-Dependent Type I Alpha Regulatory Subunit c-AMP: Cyclic Adenosine Monophosphate PKA: Protein Kinase A

Table 2 shows a classification of the cited references, offering insights into the topics covered by each study, their categorization in terms of histopathology, human samples, or clinical details, and their relevance to this study.

**Table 2:** Classification of cited references

Study Ref.	Topic - Genes/TFs/Signaling Pathways	Categorization of Studies (Study involving)	Relevance to this study
[1]	Molecular Basis of Cardiac Myxomas	Histopathology, Clinical Details	Focuses on cardiac myxoma (CM), the most prevalent benign cardiac tumors.
[2]	Cardiac Organoids	Human Samples	Addresses the limited regenerative capacity of the adult human heart, a key consideration in cardiac diseases.
[3]	Cardiac Regeneration	Histopathology	Explores cardiac regeneration, which is directly related to the study’s focus on the limited regenerative potential of cardiomyocytes in cardiac myxoma (CM) development
[4]	Cardiomyogenesis	Histopathology	Examines cardiomyogenesis, providing insights into the differentiation processes of cardiomyocytes that are crucial for understanding cardiac myxoma (CM) development.
[5]	Cardiac regeneration and repair	Histopathology	Investigates cardiac regeneration and repair, which is relevant to the study as it delves into the regenerative potential of cardiac cells, a key aspect in understanding cardiac myxoma (CM) and its implications for cardiomyocyte biology.
[6]	Cardiomyocyte cell-cycle arrest	Histopathology	Explores cardiomyocyte cell-cycle arrest, a critical concept to grasp the limited regenerative potential of cardiomyocytes
[19]	Primary Cardiac Tumors	Clinical Details	Provides insights into primary cardiac tumors, which is relevant to understanding the rarity of benign cardiac myxoma (CM) and their unique features.
[7]	Heart regeneration	Histopathology	Discusses the challenges related to heart regeneration and the limited regenerative capacity of cardiomyocytes, which is crucial for understanding the significance of cardiac myxoma (CM).
[8]	Cardiac Stem Cells	Histopathology	Explores the relevance of cardiac stem cells, which play a role in understanding cardiac regeneration and potential connections to the cardiac myxoma (CM) development
[9]	Cardiac transcription factors	Histopathology	Investigates cardiac transcription factors, shedding light on how they may influence cardiomyocyte biology and their resistance to neoplastic transformations, possibly contributing to the benign nature of cardiac myxoma (CM)
[16]	Cardiac transcription factors	Human Samples	Investigates the implications of cardiac transcription factors, shedding light on cardiac myxoma (CM) development and its relationship with cardiomyocyte biology
[10]	Cardiac Development and Remodeling	Histopathology	Explores cardiac development and remodeling, potentially offering insights into cardiac myxoma (CM) development and its implications for cardiac regeneration
[17]	Cardiac Organoids	Human Samples	Investigates cardiac organoids, providing valuable information on cardiac biology, which can aid in understanding cardiac myxoma (CM) development and its relevance to cardiac regeneration.
[18]	Cardiac Organoids	Human Samples	This provides valuable insights in unlocking the nature of cardiomyocytes
[11]	Cardiac Organoids	Human Samples	This provides valuable insights in unlocking the nature of cardiomyocytes
[12]	Cardiac Organoids	Human Samples	This provides valuable insights in unlocking the nature of cardiomyocytes
[13]	Cardiac stem cells	Human Samples	Focuses on cardiac stem cells, which are of interest due to their potential role in understanding cardiac myxoma (CM) and cardiac regeneration.
[14]	Cardiac Tumors	Clinical Details	Addresses the topic of cardiac tumors, which is relevant for understanding the etiology and characteristics of cardiac myxoma (CM)
[15]	Cardiac Tumors	Clinical Details	Explores cardiac tumorigenesis
[20]	Heart Regeneration	Human Samples	Provides insights into heart regeneration, a relevant concept for studying the limited regenerative potential of cardiomyocytes.
[21]	Cardiac Regeneration	Human Samples	Explores cardiac regenerative potential
[22]	Cardiac Regenerative Pathways	Human Samples	Essential to investigate the nature of cardiomyocytes
[23]	Cardiac Organoids	Human Samples	Provides step by step progression of the cardiac development
[24]	Cardiac Organoids	Human Samples	Provides insights into the cardiac developmental processes
[152]	Cardiac Reprogramming Factors	Histopathology	Addresses the genetic architecture involved in cardiac reprogramming
[153]	Cardiogenesis	Human Samples	Provides valuable details about the process of cardiac development
[154]	Cardiac Progenitors	Histopathology	Addresses the development of cardiomyocytes from cardiac progenitors
[155]	Heart Field	Human Samples	Provides valuable details about early stages of cardiac development
[156]	Heart Field	Histopathology	Provides valuable details about early stages of cardiac development
[58]	Heart Field	Histopathology	Provides valuable details about early stages of cardiac development
[157]	Cardiac Progenitor Cells	Human Samples	Addresses the development of cardiomyocytes from cardiac progenitors
[158]	Regenerative Cardiology	Human Samples	Helps to explore the relationship between cardiac myxoma (CM) and the limited regenerative potential of cardiomyocytes
[159]	Cardiac development and remodeling	Human Samples	Essential in understanding the mechanisms governing cardiac cell fate
[30]	Cardiogenesis	Human Samples	Essential to analyze the intricate processes of cardiac development
[25]	Islet1 - control of cardiomyocyte cell fate	Histopathology	Provides insights into Isl1 and its role in controlling cardiomyocyte cell fate, which is a key aspect of the study’s focus on cardiac myxomas and the transformation of cardiomyocytes into progenitor-like cells
[26]	Islet1 - cardiac progenitor proliferation	Histopathology	Offers insights into Isl1 and its role in cardiac progenitor proliferation
[27]	Islet1 - Cardiac Progenitor Cells	Human Samples	Focuses on Isl1 as one of the earliest expressed transcription factors in Cardiac Progenitor Cells
[28]	Islet1 - Cardiomyogenesis	Histopathology	Discusses Isl1 and its role in cardiomyogenesis, contributing to the understanding of cardiac development
[29]	Islet1 - Cardiac Development	Human Samples	Provides insights into Isl1 and its role in controlling cardiomyocyte cell fate
[31]	Islet1 and Tbx5 interaction	Human Samples	Provides insights into how these interactions may influence cardiac programming
[32]	Islet1 in Cardiac Progenitors	Human Samples	Points to the significance of Isl1 in cardiac progenitor cells
[33]	Cardiac Stem Cells	Histopathology	Aligns with the study’s exploration of cardiac myxomas and their potential influence on the limited regenerative capacity of cardiomyocytes
[34]	Islet1 – Cardiac Differentiation	Histopathology	Provides insights into the influence of Isl1 on the process of cardiac differentiation
[35]	Islet1 – Cardiac Repair	Histopathology	Provides insights into the influence of Isl1 on the process of cardiac repair
[36]	Islet1 and Nkx2–5 interaction	Histopathology	Provides insights about how these interactions may influence cardiac programming
[37]	Islet1 with Mef2c and GATA4	Histopathology	Provides insights about how these interactions may influence cardiac programming
[38]	Islet1 with Nkx2–5 and GATA4	Histopathology	Provides insights about how these interactions may influence cardiac programming
[39]	Islet1 - Cardiac Morphogenesis	Histopathology	Offers insights into the factors influencing cardiac genetic architecture
[160]	Islet1 in Cancer Progression	Human Samples	Essential to understand the nature and impact of Isl1 in tumorigenesis
[161]	Islet1 in different tumors	Human Samples	Essential to understand the nature and impact of Isl1 in tumorigenesis
[162]	Islet1 in different tumors	Human Samples	Essential to understand the nature and impact of Isl1 in tumorigenesis
[163]	Islet1 in tumorigenesis	Human Samples	Essential to understand the nature and impact of Isl1 in tumorigenesis
[164]	Islet1 in different tumors	Histopathology	Essential to understand the nature and impact of Isl1 in tumorigenesis
[40]	Multipotent cardiac stem cells	Human Samples	Essential to explore the aspects of cardiac regeneration and differentiation
[41]	Nkx2–5 and Isl1 interaction	Histopathology	Provides insights about how these interactions may influence cardiac programming
[42]	Isl1 repression	Histopathology	Related to the study’s interest in the control of cardiomyocyte cell fate and differentiation.
[43]	Islet-1 modulating estrogen receptor	Histopathology	Provides insights about how these interactions may influence cardiac programming
[44]	Islet-1 modulating estrogen receptor	Histopathology	Provides insights about how these interactions may influence cardiac programming
[45]	Islet1 and GATA4 interaction	Histopathology	Provides insights about how these interactions may influence cardiac programming
[46]	Brg1/Baf60 – Smarcd3 complex in Pluripotency and Differentiation	Histopathology	Relevant to understanding the molecular mechanisms related to cardiomyocyte reprogramming in cardiac myxoma (CM)
[47]	Brg1/Baf60 – Smarcd3 complex in Cardiac Development	Human Samples	Provides insights into the regulatory pathways that may be involved in cardiac myxoma (CM) development
[48]	Brg1/Baf60 – Smarcd3 complex in Development and Cancers	Human Samples	Potentially offering valuable information about how it may play role in the transformation of cardiomyocytes into progenitor-like cells in cardiac myxoma (CM) development
[49]	Brg1/Baf60 – Smarcd3 complex in EMT transition	Human Samples	Essential to investigate the progenitor-like state in cardiac myxoma (CM)
[50]	Brg1/Baf60 – Smarcd3 complex in EMT transition	Human Samples	Essential to investigate the progenitor-like state in cardiac myxoma (CM)
[51]	Brg1/Baf60 – Smarcd3 complex and p53 interaction	Histopathology	Addresses how key genes are regulated in cardiac development
[52]	Brg1/Baf60 – Smarcd3 complex in cardiomyocyte fate	Histopathology	Provides insights into the development of cardiogenic cell fate
[53]	Brg1/Baf60 – Smarcd3 complex in cardiac progenitor cells	Histopathology	Relevant to understanding how these cells may influence the development of cardiac myxoma and the reversion of cardiomyocytes to a progenitor-like state
[54]	Brg1/Baf60 – Smarcd3 complex in cardiogenesis	Histopathology	Provides insights into the regulatory pathways that may be involved in cardiac myxoma (CM) development
[55]	Brg1/Baf60 – Smarcd3 complex: Tbx5 in cardiogenesis	Histopathology	Provides insights about how these interactions may influence cardiac programming
[165]	Brg1/Baf60 – Smarcd3 complex in development processes	Histopathology	Provides insights about the nature of this complex
[166]	Brg1/Baf60 – Smarcd3 complex in tumorigenesis	Histopathology	Essential to understand the nature and impact of Baf60 in tumorigenesis
[56]	Brg1/Baf60 – Smarcd3 complex’s interaction with Nkx2–5	Human Samples	Provides insights about how these interactions may influence cardiac programming
[57]	Brg1/Baf60 – Smarcd3 complex’s interaction with Nkx2–5	Histopathology	Provides insights about how these interactions may influence cardiac programming
[58]	Nkx2–5 in heart field	Histopathology	Provide insights into the potential role of Nkx2–5 in cardiac myxoma development and its control over cardiac progenitor cells.
[167]	Nkx2–5 in controlling proliferation	Histopathology	Relevant to elucidate Nkx2–5’s potential role in limiting tumorigenesis in cardiac myxomas by controlling the proliferation of cardiac progenitor cells
[59]	Nkx2–5 and its interaction with wnt pathway	Histopathology	Provides insights about how these interactions may influence cardiac programming
[60]	Nkx2–5 and wnt in cardiogenesis	Histopathology	Provides insights about how these interactions may influence cardiac programming
[61]	Nkx2–5 as tumor suppressor	Histopathology	Provides insights into how Nkx2–5 exerts influence over the risk of cardiac tumorigenesis
[62]	Nkx2–5 in cardiogenesis	Histopathology	Potentially shedding light on its influence in cardiac myxoma development
[63]	Nkx2–5 and its interactions with Tbx1 in cardiogenesis	Histopathology	Provides insights about how these interactions may influence cardiac programming
[64]	Interaction of Nkx2–5 with tumor suppressor genes	Human Samples	Provides insights about how these interactions may influence cardiac programming
[65]	Nkx2–5 in cardiac differentiation	Histopathology	Provides insights into how Nkx2–5 exerts influence over the risk of cardiac tumorigenesis
[66]	Nkx2–5 and its interaction with BMP signaling	Histopathology	Provides insights about how these interactions may influence cardiac programming
[67]	Nkx2–5 and its interaction with FGF	Histopathology	Provides insights about how these interactions may influence cardiac programming
[68]	Nkx2–5 and its interactions with cardiac regulator genes	Histopathology	Provides insights about how these interactions may influence cardiac programming
[168]	Nkx2–5 in tumors	Human Samples	Essential to understand the nature and impact of Nkx2–5 in tumorigenesis
[169]	Nkx2–5 in tumors	Human Samples	Essential to understand the nature and impact of Nkx2–5 in tumorigenesis
[170]	Nkx2–5 and its interaction with tumor suppressor gene	Histopathology	Provides insights about how these interactions may influence cardiac programming
[171]	Nkx2–5 in tumors	Human Samples	Essential to understand the nature and impact of Nkx2–5 in tumorigenesis
[172]	Nkx2–5 in tumors	Human Samples	Essential to understand the nature and impact of Nkx2–5 in tumorigenesis
[173]	Nkx2–5 in tumors	Human Samples	Essential to understand the nature and impact of Nkx2–5 in tumorigenesis
[174]	Nkx2–5 in tumors	Histopathology and Clinical Details	Essential to understand the nature and impact of Nkx2–5 in tumorigenesis
[69]	Transcription factors in Cardiac Myxoma	Human Samples	Explores transcription factors in cardiac myxoma, which aligns with the focus of this study on cardiac myxoma and the role of transcription factors in its development
[70]	Nkx2–5 in cardiogenesis	Histopathology	Potentially shedding light on its influence in cardiac myxoma development
[71]	Nkx2–5 and Oct4: cardiac reprogramming	Histopathology	Provides insights about how these interactions may influence cardiac programming
[72]	Nkx2–5 and Oct4: cardiac reprogramming	Human Samples	Provides insights about how these interactions may influence cardiac programming
[73]	GATA4 in cardiogenesis	Histopathology	Potentially shedding light on its influence in cardiac myxoma development
[74]	GATA4 in cardiogenic potential	Histopathology	Potentially shedding light on its influence in cardiac myxoma development
[75]	GATA4 and its interaction with cyclin D2	Human Samples	Provides insights about how these interactions may influence cardiac programming
[76]	GATA4 and Shh in cardiogenesis	Histopathology	Provides insights about how these interactions may influence cardiac programming
[77]	GATA4 as tumor suppressor	Human Samples	Essential to understand the benign nature of cardiac myxoma
[78]	GATA4 as tumor suppressor	Human Samples	Essential to understand the benign nature of cardiac myxoma
[79]	GATA4 as tumor suppressor	Human Samples	Essential to understand the benign nature of cardiac myxoma
[175]	GATA4 in cardiac repair	Human Samples	To understand the scope of GATA4 in cardiac programming
[176]	GATA4 in cardiac remodeling	Human Samples	To understand the scope of GATA4 in cardiac programming
[177]	GATA4 in activating cardiac gene expression	Histopathology	To understand the scope of GATA4 in cardiac programming
[178]	GATA4 and its tumor suppressor role	Histopathology	Essential to understand the benign nature of cardiac myxoma
[179]	GATA4 in liver tumors	Human Samples	Essential to understand the nature and impact of GATA4 in tumorigenesis
[180]	GATA4 in other tumors	Human Samples	Essential to understand the nature and impact of GATA4 in tumorigenesis
[181]	GATA4 in other tumors	Human Samples	Essential to understand the nature and impact of GATA4 in tumorigenesis
[182]	GATA4 and its interaction with Wnt pathway	Histopathology	Provides insights about how these interactions may influence cardiac programming
[80]	GATA4 in cardiac tumorigenesis	Clinical Details	Relevant to the study’s focus on understanding the origins and mechanisms underlying cardiac myxoma development.
[81]	GATA4 in cardiac tumorigenesis	Histopathology	Relevant to the study’s focus on understanding the origins and mechanisms underlying cardiac myxoma development.
[82]	Tbx5 in stem cells	Human Samples	Aligns with the study’s exploration of Tbx5 and other key cardiac genes/TFs in the context of cardiomyocyte biology and cardiac regeneration
[83]	Tbx5 in stem cells	Human Samples	Aligns with the study’s exploration of Tbx5 and other key cardiac genes/TFs in the context of cardiomyocyte biology and cardiac regeneration
[84]	Tbx5 in cardiogenesis	Histopathology	Aligns with the study’s exploration of Tbx5 and other key cardiac genes/TFs in the context of cardiomyocyte biology and cardiac regeneration
[85]	Mutated Tbx5	Histopathology	To understand the extent of Tbx5 role in governing cardiomyocytes
[86]	Tbx5 in cardiac cell fate	Histopathology	Provides insights about the nature of cardiomyocytes
[87]	Tbx5 and its interaction with mef2c	Histopathology	Provides insights about how these interactions may influence cardiac programming
[88]	Tbx5 and its role as tumor suppressor	Human Samples	Essential to understand the benign nature of cardiac myxoma
[89]	Tbx5 and its dysregulations in cardiogenesis	Histopathology	To understand the extent of Tbx5 role in governing cardiomyocytes
[90]	Mutated Tbx5	Histopathology	To understand the extent of Tbx5 role in governing cardiomyocytes
[91]	Mutated Tbx5	Histopathology	To understand the extent of Tbx5 role in governing cardiomyocytes
[92]	Tbx5 in cardiac differentiation	Histopathology	Provides insights about the limited regenerative capacity of cardiac cells and rare cardiac tumorigenesis
[93]	Tbx5 in cardiac differentiation	Histopathology	Provides insights about the limited regenerative capacity of cardiac cells and rare cardiac tumorigenesis
[94]	Tbx5 in promoting cardiac cell fate	Histopathology	Relevant to understand the role of Tbx5 in cardiac homeostasis
[183]	Tbx5 in cardiac developmental defects	Histopathology	To understand the extent of Tbx5 role in governing cardiomyocytes
[184]	Tbx5 in other tumors	Human Samples	Relevant to the study’s focus on understanding the origins and mechanisms underlying cardiac myxoma development.
[185]	Tbx5 as tumor suppressor	Human Samples	Essential to understand the benign nature of cardiac myxoma
[186]	Tbx5 in other tumors	Human Samples	Relevant to the study’s focus on understanding the origins and mechanisms underlying cardiac myxoma development.
[95]	Tbx5 in inducing cardiac cell fate	Human Samples	Provides insights about the nature of cardiomyocytes
[96]	Tbx5 in cardiac development	Histopathology	Provides insights about the nature of cardiomyocytes
[97]	Mef2c in cardiogenesis	Histopathology	Relevant to understand the proliferative cardiac signaling
[98]	Mef2c in myocardium	Histopathology	Related to interactions with proliferative genes
[99]	Mef2c and its interaction with Nkx2–5	Histopathology	Provides insights about how these interactions may influence cardiac programming
[100]	Mef2c in cardiac development	Histopathology	Potentially shedding light on its influence in cardiac myxoma development
[101]	Mef2c and its interaction with Nkx2–5	Histopathology	Provides insights about how these interactions may influence cardiac programming
[187]	Mef2c in other tumors	Clinical Details	Relevant to the study’s focus on understanding the origins and mechanisms underlying cardiac myxoma development.
[188]	Mef2c in other tumors	Histopathology	Relevant to the study’s focus on understanding the origins and mechanisms underlying cardiac myxoma development.
[189]	Mef2c and its interactions with stemness regulators	Histopathology	Provides insights about progenitor-like state in cardiac myxoma
[190]	Mef2c in other tumors	Histopathology	Essential to understand the its nature and impact on tumorigenesis
[191]	Mef2b in other tumors	Human Samples	Essential to understand the its nature and impact on tumorigenesis
[192]	Mef2b in other tumors	Human Samples	Essential to understand the its nature and impact on tumorigenesis
[102]	Mef2c in cardiac tumorigenesis	Histopathology	Essential to understand the its nature and impact on tumorigenesis
[103]	Mef2c in cardiogenesis	Histopathology	Relevant to cardiac genetic programming
[104]	Mef2c in cardiogenesis	Histopathology	Relevant to cardiac genetic programming
[105]	Mef2c and its interaction with Tbx1	Histopathology	Provides insights about how these interactions may influence cardiac programming
[106]	HAND1/2 in cardiac differentiation	Human Samples	Provides insights about the limited regenerative capacity of cardiac cells and rare cardiac tumorigenesis
[107]	HAND1/2 in cardiogenesis and in heart defects	Histopathology	Relevant to understand its impact on cardiac programing and in different stages of cardiac development
[108]	HAND1/2 in cardiac regeneration	Histopathology	Relevant to understand its impact on cardiac programing and on the limited cardiac regenerative potential
[109]	HAND1/2 in cardiac regeneration	Histopathology	Relevant to understand its impact on cardiac programing and on the limited cardiac regenerative potential
[193]	HAND1/2 in other tumors	Histopathology	Essential to understand the its nature and impact on tumorigenesis
[194]	HAND1/2 as tumor suppressor	Human Samples	Provides insights about rare occurrence of cardiac tumors
[195]	HAND1/2 as tumor suppressor	Histopathology	Provides insights about rare occurrence of cardiac tumors
[196]	HAND1/2 as tumor suppressor	Human Samples	Provides insights about rare occurrence of cardiac tumors
[110]	HAND1/2: Cardiac myxoma showing progenitor-like state	Human Samples	Relevant to understand how HAND1/2 govern the cardiac fate and prevent cardiomyocytes from reverting to progenitor-like state, hallmark of cardiac myxoma
[111]	MYOCD - Cardiogenesis	Histopathology	Aligns with the study’s objective of understanding how MYOCD influences cardiomyocyte biology
[112]	MYOCD in different cell types including cardiomyocytes	Human Samples	Provides insights about different roles it plays in different microenvironments
[113]	MYOCD in stem cells	Histopathology	Provides insights about its influence on the stem cells, to understand the development of progenitor-like state in cardiac myxoma
[197]	MYOCD in cardiac cell fate	Human Samples	Relevant to understand how this gene influences the combinatorial code of cardiomyocytes
[198]	MYOCD in other tumors	Human Samples	Essential to understand the its nature and impact on tumorigenesis
[199]	MYOCD as tumor suppressor	Histopathology	Provides insights about rare occurrence of cardiac tumors
[200]	MYOCD and its interactions	Histopathology	Provides insights about how these interactions may influence cardiac programming
[201]	MYOCD in different tumors	Histopathology	Essential to understand the its nature and impact on tumorigenesis
[202]	MYOCD in different tumors	Histopathology	Essential to understand the its nature and impact on tumorigenesis
[203]	MYOCD and its interactions in promoting cardiogenic potential	Human Samples	Provides insights about how these interactions may influence cardiac programming
[114]	MSX2 and its role in stemness	Histopathology	Provides insights about its influence on the stem cells, to understand the development of progenitor-like state in cardiac myxoma
[115]	MSX2 in cardiac development	Histopathology	Relevant to understand how this gene influences the combinatorial code of cardiomyocytes
[116]	MSX2 and its role in stemness	Human Samples	Provides insights about its influence on the stem cells, to understand the development of progenitor-like state in cardiac myxoma
[204]	MSX2 in promoting stemness	Human Samples	Provides insights about its influence on the stem cells, to understand the development of progenitor-like state in cardiac myxoma
[205]	MSX2 in tumorigenesis	Histopathology	Essential to understand the its nature and impact on tumorigenesis
[206]	MSX2 in other tumors	Histopathology	Essential to understand the its nature and impact on tumorigenesis
[207]	MSX2 in cardiac diseases	Histopathology	Relevant to understand how this gene influences the combinatorial code of cardiomyocytes in diseased states
[208]	MSX2 in other tumors	Human Samples	Essential to understand the its nature and impact on tumorigenesis
[209]	MSX2 and its interaction with other stemness genes	Human Samples	Provides insights about how these interactions may influence cardiac programming
[210]	MSX genes and their impact on apoptosis	Histopathology	Provides insights about rare occurrence of cardiac tumors
[117]	HOPX and its interaction with Wnt and BMPs	Histopathology	Provides insights about how these interactions may influence cardiac programming
[118]	HOPX and its interaction with Nkx2–5	Histopathology	Provides insights about how these interactions may influence cardiac programming
[119]	HOPX in cardiac differentiation	Histopathology	Provides insights about the limited regenerative capacity of cardiac cells and rare cardiac tumorigenesis
[211]	HOPX in other tumors	Human Samples	Essential to understand the its nature and impact on tumorigenesis
[212]	HOPX in other tumors	Histopathology	Essential to understand the its nature and impact on tumorigenesis
[213]	HOPX as tumor suppressor	Human Samples	Provides insights about rare occurrence of cardiac tumors
[120]	Wnt in cardiogenesis	Histopathology	Relevant to the progenitor-like state in cardiac myxoma
[121]	Wnt in cardiac development and disease	Histopathology	Relevant to the progenitor-like state in cardiac myxoma
[122]	Wnt in cardiogenesis	Histopathology	Provides insights about wnt’s impact on the cardiac proliferative potential
[123]	Wnt and its impact on cardiac differentiation	Histopathology	Provides insights about wnt’s impact on limiting the cardiac differentiation and possible impact in the development of progenitor-like state.
[214]	Wnt and its impact on apoptosis	Histopathology	Relevant to the progenitor-like state in cardiac myxoma
[215]	Wnt in different tumors	Histopathology	Essential to understand its nature and impact on tumorigenesis
[216]	Wnt in different tumors	Histopathology	Essential to understand its nature and impact on tumorigenesis
[217]	Wnt in cardiac regeneration	Histopathology	Provides insights about wnt’s impact on the cardiac regenerative potential
[218]	Wnt’s interaction with BMPs	Histopathology	Provides insights about how these interactions may influence cardiac programming
[219]	Wnt’s interaction with Isl1 and FGF	Histopathology	Provides insights about how these interactions may influence cardiac programming
[220]	Wnt in renewal of stem cells	Histopathology	Relevant to the progenitor-like state in cardiac myxoma
[221]	Wnt in dedifferentiation	Human Samples	Relevant to the progenitor-like state in cardiac myxoma
[124]	Wnt in cardiac progenitor cells	Histopathology	Relevant to the progenitor-like state in cardiac myxoma
[125]	Wnt’s interaction with NF-κB	Histopathology	Provides insights about how these interactions may influence cardiac programming
[126]	FGF in heart field	Histopathology	Provides insights about early stages of cardiogenesis
[127]	FGF in heart field	Histopathology	Provides insights about early stages of cardiogenesis
[128]	FGF in cardiogenesis, regeneration and repair	Histopathology	Relevant to understand the limited cardiac regenerative capacity and its impact on the nature of cardiac tumors
[129]	FGF in transitions involved in cardiac development	Histopathology	Relevant to understand the progenitor-like state in cardiac myxoma
[130]	FGF in cardiac differentiation	Histopathology	Provides insights about the cardiac cell-type specific programming
[131]	FGF in cardiomyocyte survival	Histopathology	Relevant to understand the homeostasis in cardiomyocytes
[132]	FGF in stem cells	Histopathology	Relevant to understand the progenitor-like state in cardiac myxoma
[222]	FGF and its interaction with BMPs in heart field	Histopathology	Provides insights about how these interactions may influence cardiac programming
[223]	FGF in postnatal cardioprotection	Histopathology	Relevant to understand the homeostasis in cardiomyocytes
[224]	FGF in preventing pathologic cardiac remodeling	Histopathology	Relevant to understand the homeostasis in cardiomyocytes
[225]	FGF in cardioprotection	Histopathology	Relevant to understand the homeostasis in cardiomyocytes
[226]	FGF and its interactions with other cardiac pathways	Histopathology	Provides insights about how these interactions may influence cardiac programming
[227]	FGF in controlling cardiomyocyte cell cycle	Histopathology	Provides insights about the limited cardiac regenerative capacity
[228]	FGF and its interaction with Nkx2–5	Human Samples	Provides insights about how these interactions may influence cardiac programming
[229]	FGF in cardiac differentiation	Human Samples	Provides insights about the cardiac cell-type specific programming
[133]	FGF expression in cardiac myxoma	Human Samples	Relevant to understand the progenitor-like state in cardiac myxoma
[134]	FGF in cardiac injury	Histopathology	Provides insights about the limited cardiac regenerative capacity
[135]	BMPs in cardiac cell fate	Histopathology	Investigates BMPs in the context of cardiac cell fate
[136]	BMPs in cardiac differentiation	Histopathology	Provides insights about the cardiac cell-type specific programming
[137]	BMPs in cardiac differentiation	Histopathology	Provides insights about the cardiac cell-type specific programming
[138]	BMPs and their interaction with Nkx2–5	Histopathology	Provides insights about how these interactions may influence cardiac programming
[139]	BMPs and their interaction with Nkx2–5	Histopathology	Provides insights about how these interactions may influence cardiac programming
[230]	BMPs in different tumors	Histopathology	Essential to understand its nature and impact on tumorigenesis
[231]	BMPs in cardiac differentiation	Histopathology	Provides insights about the cardiac cell-type specific programming
[232]	BMPs in different tumors	Histopathology	Essential to understand their nature and impact on tumorigenesis
[233]	BMPs in different tumors	Human Samples	Essential to understand their nature and impact on tumorigenesis
[234]	BMPs in mesenchymal stem cells	Human Samples	Relevant to understand the progenitor-like state in cardiac myxoma
[235]	BMPs and their role in progenitor-like state	Histopathology	Relevant to understand the progenitor-like state in cardiac myxoma
[236]	BMPs in tumorigenesis	Human Samples	Essential to understand their nature and impact on tumorigenesis
[237]	BMPs in different tumors	Histopathology	Essential to understand their nature and impact on tumorigenesis
[238]	BMPs in different tumors	Human Samples	Essential to understand their nature and impact on tumorigenesis
[239]	BMPs in promoting tumorigenesis	Human Samples	Essential to understand their nature and impact on tumorigenesis
[240]	BMPs in promoting tumorigenesis	Human Samples	Essential to understand their nature and impact on tumorigenesis
[140]	Notch in lineage commitment and in cardioprotection	Histopathology	Provides insights about the cardiac cell-type specific programming and about the nature of cardiomyocytes
[141]	Notch in cardiac regeneration	Histopathology	Provides insights about the limited cardiac regenerative potential
[142]	Notch in development	Histopathology	Provides insights about the cardiac cell-type specific programming
[143]	Notch in cardiogenesis	Histopathology	Relevant to understand the early stages of cardiac development
[144]	Notch overexpression in oncogenic transformation	Histopathology	Essential to understand its nature and impact on tumorigenesis
[145]	Notch and its interaction with BMPs in cardiogenesis	Histopathology	Provides insights about how these interactions may influence cardiac programming
[146]	Notch signaling in cardiac development and disease	Histopathology	Provides insights about the cardiac cell-type specification in different states
[241]	Notch in promoting stemness	Human Samples	Provides insights about the progenitor-like state in cardiac myxoma
[242]	Notch in controlling the maintenance and commitment of cardiac stem cells	Histopathology	Provides insights about the cardiac cell-type specific programming
[243]	Notch in stemness and tumorigenesis	Human Samples	Provides insights about the progenitor-like state in cardiac myxoma
[244]	Notch and its interaction with Wnt pathway in regulating stemness	Human Samples	Provides insights about how these interactions may influence cardiac programming and contribute to the development of progenitor-like state in cardiac myxoma
[148]	Clinical and molecular features of the Carney complex	Histopathology	Relevant to understand the nature of cardiac myxoma
[149]	PRKAR1A – Carney Complex Mutations	Human Samples	Relevant to understand the nature of cardiac myxoma
[147]	Cardiac myxoma in Carney complex	Clinical Details	Relevant to understand the nature of cardiac myxoma
[150]	Genotype-phenotype correlation for PRKAR1A mutations	Human Samples	Relevant to understand the nature of cardiac myxoma
[151]	Cardiac myxomas in Carney complex	Clinical Details	Relevant to understand the nature of cardiac myxoma
[245]	Endothelial-cardiomyocyte crosstalk in cardioprotection	Histopathology	Relevant to understand the cardiac homeostasis
[246]	Islet1 and GATA4 in cardiac regeneration	Histopathology	Essential in understanding the interactions among the proliferation and differentiation-related genes to understand the genetic landscape of cardiomyocytes
[247]	Cell Generation and Turnover in the Human Heart	Human Samples	Relevant to understand the cardiac homeostasis
[248]	Decline in cardiac regenerative potential	Histopathology	Provides insights about the limited cardiac regenerative potential
[249]	Cardiac gene regulatory programs	Human Samples	Provides insights about the cardiac cell-type specific genetic programming
[250]	Cardiogenesis and cardiac heart defects	Histopathology	Relevant to understand the significance of cardiac cell-type specific genetic programming
[251]	Cardiac cell fate	Human Samples	Provides insights about the cardiac cell-type specific genetic programming
[252]	Microenvironment in cardiac tumor development	Histopathology	Provides insights about the cardiac tumorigenesis
[253]	Cardiac Stem Cell Senescence	Histopathology	Provides insights about the impact of cardiac aging on cardiac cell-type specific genetic programming
[254]	Cardiac Stem Cell Aging	Histopathology	Provides insights about the impact of cardiac aging on cardiac cell-type specific genetic programming
[255]	Regeneration of the aging cardiovascular system	Histopathology	Provides insights about the impact of cardiac aging on cardiac cell-type specific genetic programming
[256]	Gene expression of cancers and its relationship to other diseases	Histopathology	Provides insights about the relationship among different genes involved in tumorigenesis landscape and how they are affected in cell types with limited regenerative capacity
[257]	Cardiac aging	Histopathology	Provides insights about the impact of cardiac aging on cardiac cell-type specific genetic programming
[258]	Cardiac Stem Cells in the Postnatal Heart	Histopathology	Provides insights about cardiac homeostasis
[259]	Cardiac regenerative potential	Histopathology	Relevant to understand the limited cardiac regenerative capacity
[260]	Tumor heterogeneity	Histopathology	Provides insights about tumorigenesis
[261]	Tumor heterogeneity	Histopathology	Provides insights about tumorigenesis
[262]	Tumor heterogeneity and its relation to plasticity	Histopathology	Provides insights about tumorigenesis
[263]	Tumor heterogeneity	Histopathology	Provides insights about tumorigenesis
[264]	Cardiogenesis in congenital heart disease	Histopathology	Provides insights about the cardiac cell-type specific genetic programming in cardiac defects
[265]	Cardiogenesis in congenital heart disease	Histopathology	Provides insights about the cardiac cell-type specific genetic programming in cardiac defects
[152]	Cardiac Reprogramming	Histopathology	Provides insights about the cardiac cell-type specific genetic programming
[266]	Cardiogenesis	Histopathology	Provides insights about the cardiac cell-type specific genetic programming
[267]	Cardiac gene-editing	Histopathology	Provides insights about the new potential therapeutic targets to tackle cardiac diseases
[268]	Gene-editing strategies in cardiovascular cells	Histopathology	Provides insights about the new potential therapeutic targets to tackle cardiac diseases
[269]	Gene-editing in harnessing cardiac regenerative potential	Histopathology	Provides insights about the new potential therapeutic targets to tackle cardiac diseases
[270]	Gene-editing and cardiovascular disease	Histopathology	Provides insights about the new potential therapeutic targets to tackle cardiac diseases
[271]	Gene-editing in cardiac research	Histopathology	Provides insights about the new potential therapeutic targets to tackle cardiac diseases
[272]	Cardiac regeneration	Histopathology	Provides insights about the cardiac cell-type specific genetic programming in relation to cardiac regenerative potential

## Key cardiac transcription factors/genes

In this section, this study investigates cardiac myxoma through the lens of developmental biology [152, 153]. The CPCs are multi-lineage cells with major expression of *Nkx2–5* and *Isl1* [154, 155]. By increasing the gene expressions of *BMP* signaling pathway and downregulating *Wnt* pathway, the CPCs begin to differentiate into cardiomyocytes [156]. The *Isl1* and *Nkx2–5* TFs act via activating cascade of downstream cardiac genes in time specific manner [58].

It is important to note that *Isl1* is a pioneering Transcription factor (PTF) of cardiomyocyte cell fate. As *Isl1* expression begins to decline, the *HOPX* becomes upregulated [157]. The *Nkx2–5* has very strong interactions with HOPX as it acts as a downstream regulator of *HOPX* and governs its gene expression. The *HOPX* is expressed in cardiomyoblast and is very important in the process of differentiation as it is also expressed in pre-cardiac mesoderm. *HOPX* positively interacts with *BMPs, SMADs* and negatively with *Wnt*-signaling and *Axin2* signaling pathway. The *Wnt*-pathway and *Axin2* oppose differentiation of CPCs [158].

In CPCs, the *Nkx2–5* expression continuously increases over the duration of differentiation. When *HOPX* is defective, *Wnt*-pathway becomes upregulated and this downregulates *Nkx2–5*. Normally, the *BMP-SMAD* complex is activated by *HOPX*. This downregulates *WNT*-signaling pathway and promotes differentiation of CPCs towards cardiomyocyte development. This *BMP-SMAD* complex increases *MSX1* expression to promote differentiation and downregulates *Axin2* [159].

### Islet1

#### Role in cardiogenesis

*Islet1 (Isl1)* plays the role of a PTF in epigenetic control of cardiomyocyte cell fate [25]. This also governs epigenetic programming and shapes chromatin landscape. It works with additional regulatory factors to specify cell lineage and cardiac differentiation [26]. *Isl1* governs a regulatory network of genes that is involved in unfolding cardiac lineage [27]. It is transiently expressed in CPCs including atrial area and is involved in their proliferation, survival and migration [28]. When *Isl1* is defective, cardiac development gets disrupted. *Isl1* expression is greater before progenitor cells differentiate into heart tube [29].

#### Proliferation-related roles

*Isl1* is one of the earliest genes expressed in the cardiac progenitors. *Isl1* interacts strongly with *Tbx1* and both are strongly expressed in multipotent CPCs [30, 31]. It also has major interaction with *Wnt-*signaling pathway. *Isl1* also works with *FGF10* to promote proliferation of progenitor cells [32]. *Isl1* expression also plays important roles in the neural crest cells, and in heart, in which the progenitor cells expressing *Isl1* are capable of differentiating into cardiomyocytes, endothelial and smooth muscle lineage [33]. The CPCs also maintain expression of *Isl1* in postnatal state. The *Isl1* expression is essential in renewal of cardiac progenitors. Prior to differentiation, the gene expression of *Isl1* contributes to proliferation of CPCs [34, 35].

#### Key interactions with tumor suppressors/ differentiation-related genes

*Isl1* sets in motion the gene expression of *Brg1- Baf60* which contributes to commit CPCs towards cardiomyocyte fate. The cardiac differentiation-related genes downregulate the expression of genes involved in the maintenance of progenitor-like state in CPCs. *Nkx2–5* promotes the process of differentiation, and downregulates the gene expression of *Isl1* [36]. *GATA4* activates *Isl1* enhancer. The *BMPs* downregulate the gene expression of *Isl1* and *Tbx1* to increase myocardial differentiation [37].

#### Contributions to combinatorial code/ cell type specific genetic-programming

The induction of cell type specific genetic-programming needs PTFs such as *Isl1* which works with other special TFs to form combinations which determine the final cell type and regulate the process of cardiomyocyte development [38, 39].

#### Presence in other tumors

Upregulated expression of *Isl1* is present in many cancers including pheochromocytoma, pancreatic, gastrointestinal, lung tumors, bile duct carcinoma, prostate and breast cancers. *Isl1* expression is also present in insulinoma cells, bladder cancer, Non-Hodgkin lymphoma, glioma, melanoma and others [160–163]. *Isl1* is a novel regulator of *cyclins* and *c-myc* gene. This also emphasizes the role of Isl1 in tumor development [164].

#### Possible role in cardiac myxoma

Despite being very similar to multipotent CPCs, CM are *c-kit* positive but very rarely *Isl1* positive [40]. As the *Nkx2–5* has been found to be involved in CM development, its role in interacting with *Isl1* is very significant. Possibly, the influence of *Nkx2–5* over the genetic landscape in cardiac myxoma prevents the progression of cardiac myxoma cells towards malignant state [41, 42].

*Isl1* and *GATA4* also interact with Estrogen Receptor alpha. This may be a contributing factor in the development of more cardiac myxoma cases in female patients [43–45].

#### Summary: unraveling the multifaceted roles of Isl1

Isl1 is a pivotal player in cardiogenesis, orchestrating cardiomyocyte cell fate by shaping the epigenetic landscape and interacting with regulatory factors. It forms a network of genes that guide cardiac lineage development, with transient expression in cardiac progenitor cells, influencing proliferation, survival, and migration. Malfunctions in Isl1 disrupt cardiac development. It interacts strongly with Tbx1, the Wnt pathway, and FGF10 to promote progenitor cell proliferation and differentiation into various cardiac cell types. Isl1 also influences cardiomyocyte commitment by partnering with Brg1-Baf60. Its interactions with differentiation-related genes such as Nkx2–5, GATA4, and BMPs, drive myocardial differentiation. Isl1 collaborates with other transcription factors to determine final cell types, and its upregulated expression is found in various cancers. Despite its presence in multipotent cardiac progenitor cells, Isl1 is rarely expressed in cardiac myxomas, possibly influenced by Nkx2–5, GATA4, and Estrogen Receptor alpha interactions.

### Brg1/Baf60 – Smarcd3 complex

#### Role in cardiogenesis

It is massively expressed in early stage of cardiac development [46]. Its defects exhibit cardiac morphogenetic defects as it acts as transcriptional regulator. It promotes progenitors towards cardiomyocytes, whereas its over-expression has been found to accelerate the activation of cardiac lineage-related target genes [47, 48].

#### Proliferation-related roles

When this complex is under the influence of *Wnt*-signaling, it contributes to epithelial-mesenchymal transition (EMT). The *Baf60* is capable of inducing proliferation in progenitor cells, but with *SMARCD3* complex its function becomes so much different and it contributes to cardiac differentiation. In neural progenitors, it interacts with notch to promote proliferation [49, 50].

#### Key interactions with tumor suppressors/ differentiation-related genes

It mediates interactions with core cardiac TFs including *Tbx5, Nkx2–5* and *GATA4*. Specifically, it promotes the binding of *GATA4 and Tbx5* to cardiac specific genes, thus inducing downstream regulatory networks. The process of cardiac differentiation becomes defective when there are defects in this complex [51]. *SMARCD3* is also considered to play a TS role [52, 53].

#### Contributions to combinatorial code/ cell type specific genetic-programming

This complex when combined with *GATA4* and *Tbx5*, is capable of switching on the cardiac gene expression in non-cardiac regions. It is capable of driving mesenchymal cells to develop into cardiomyocytes. This complex has major interactions with *GATA4* to turn on cardiac-specific gene expression [54] Then it combines with *Tbx5* to repress the gene expression of non-cardiac genes. This complex has considerable control over cardiac differentiation and may have fundamental influence over cardiac regeneration potential, as it is one of the key parts of cardiac-specific cell programming [55].

#### Presence in other tumors

*Brg1/Baf60 – Smarcd3* complex plays multiple roles in different cancers depending on the microenvironment and also influenced of predominant signaling pathways such as *Wnt, TGF- beta and MAPK*. In colorectal cancer, it works with *Wnt*-pathway to promote metastasis. On the contrary, it acts as a possible TS in breast cancer [56, 57, 165, 166].

#### Possible role in cardiac myxoma

There is not much data about the role of this complex in CM. The role that this gene complex plays is variable and is also dependent on the gene expression of other key regulatory genes/TFs and signaling pathways. *Wnt*-signaling is involved in the proliferation of progenitor cells, but in the presence of *GATA4* and *Tbx5* it promotes the differentiation of cardiomyocytes. *Baf60* complex works by interacting with genes/TFs involved in governing the gene expression of CPCs. Similarly, in CM development the expression of *Baf60* complex may promote tumorigenesis because of the dysregulated expression of genes/TFs involved in progenitor-like state, that is hallmark of CM.

#### Summary: unraveling the multifaceted roles of Brg1/Baf60-Smarcd3 complex

The Brg1/Baf60-Smarcd3 complex plays a pivotal role in cardiogenesis by acting as a transcriptional regulator expressed early in cardiac development. It promotes progenitors’ transition to cardiomyocytes, and its over-expression accelerates the activation of cardiac lineage-related genes. When influenced by Wnt-signaling, it contributes to epithelial-mesenchymal transition (EMT) and cardiac differentiation, interacting with key regulators like Tbx5, Nkx2–5, and GATA4. This complex can reprogram non-cardiac regions into cardiac gene expression, affecting cardiac-specific cell programming. In various cancers, its roles vary depending on microenvironment and signaling pathways. While little is known about its role in cardiac myxoma, its interactions with regulatory genes and signaling pathways may influence tumorigenesis by affecting progenitor-like states, a hallmark of CM.

### Nkx2–5

#### Role in cardiogenesis

The TF *Nkx2–5* is among the very first cardiac specific patterning genes in cardiac development. The site of its expression, the heart forming region plays key roles in cardiac specification, differentiation and proliferation [56]. It is one of the four key regulators of cardiac cell type. It is expressed in precursor cardiac cells and leads to proper cardiac development. It decides atrial and ventricular fate and defects in the gene can lead to congenital heart defects [57].

#### Proliferation-related roles

This gene is of key significance as it results in a cascade of downstream signaling and induces the cardiac programming in pluripotent mesenchymal stem cells. It is dependent on *JAK-STAT* pathway. The TF *Nkx2–5* controls cardiomyocyte differentiation by working with *Mef2c*, which is a key enhancer of *Nkx2–5* [58]. The most significant aspect of this TF related to the etiology of cardiac myxomas is that *Nkx2–5* is first expressed in CPCs and its gene expression is downregulated temporarily during cardiomyocyte differentiation [167]. However, a constant low level of *Nkx2–5* gene expression persists throughout life. It is involved in the induction of initial but not late phases of cardiomyocyte development [59]. Moreover, it interacts with notch signaling pathway to promote proliferation of CPCs. The final determination of cardiovascular lineages is regulated by *Nkx2–5* in the earliest specified multipotent cardiac progenitors. Early multipotent cardiovascular progenitor cells expressing *Nkx2–5* give rise to endothelial lineages, smooth muscles cells and cardiomyocytes [60].

#### Key interactions with tumor suppressors/ differentiation-related genes

*Nkx2–5* has major interactions with *GATA4* and *Tbx5*. It establishes a positive feedback loop with *GATA4* and interacts with *Tbx5* to enhance the differentiation of CPCs into cardiomyocytes. It directs cardiac looping by working with *MEF2c, Hand1* and *Hand2* [61]. Working with *Nkx2–7, Nkx2–5* is involved in maintenance of cardiomyocyte cellular identity. The expression of *Nkx2–5* is very significant during cardiomyocyte differentiation, as it acts as a repressor of *FGF10* and *Isl1* to enhance differentiation [62]. *Nkx2–5* enhances cardiac phenotype by antagonizing *TBX1* which is involved in the proliferation of CPCs [63]. *Nkx2–5* interacts with *BMP* signaling pathway to enhance differentiation. As *Nkx2–5* is a cardiac-specific patterning TF, its expression represses non-cardiac genes while inducing the gene expression of cardiogenic genes. The presence of *Nkx2–5* expression sets controls in cardiac cells that give them cell type-specific features such as the permanent nature of cardiac cell type. TF *Nkx2–5*, working as master regulator of cardiac development, induces a cascade of regulatory and developmental genes [64]. This sets in motion the cell type-specific combinatorial code that governs cardiomyogenesis. In post-natal cardiomyocytes, it is needed for proper functioning. *NPCEDRG* is a novel tumor suppressive gene and has potential binding sites for *Nkx2–5,* thus inducing cell differentiation, controlling cell growth and regulating the cell cycle. *Nkx2–5* influences and regulates gene activity of *HOPX* to modulate cardiac gene expression. *HOPX* has a potential tumor suppressive activity [65].

#### Contributions to combinatorial code/ cell type specific genetic-programming

*Nkx2–5* sets in motion a cascade of combinatorial genetic interactions that govern the genetic programming of cardiogenesis. The *Nkx2–5* based early patterning sets stage for BMP and Notch based gene expression. The defects in *Nkx2–5* expression down-regulate *BMPs* and *Notch* signaling pathway, resulting in the disruption of cardiogenesis [66]. TF *Nkx2–5* auto-regulates itself and is further mainly regulated by *GATA4* and *SMAD* proteins. Its expression is also dependent on *Isl1*. In the induction of the TF *Nkx2–5*, the expression of *BMP2/4* is required and the activity of *Wnt*-pathway is inhibited. The *Wnt-*signaling negatively impacts the TF *Nkx2–5* gene expression, hence must be inhibited. The *BMPs* induces the *FGF8* signaling to promote the development of cardiac proteins. *Nkx2–5* is involved in the upregulation of *HAND1* and *HAND2*, resulting in the differentiation and proliferation of cardiac cells [67]. The TF *Nkx2–5* also has major interactions with *p53, FGF16 and FGF10*. Through its interactions, it controls proliferation and differentiation [68].

#### Presence in other tumors

The role of *Nkx2–5* varies in different microenvironments; it may promote both proliferation and differentiation in different situations. *Nkx2–5* is dysregulated in acute lymphoblastic leukemia (ALL), hepatocellular carcinoma (HCC), and T cell neoplasias, methylated in prostate adenocarcinoma, hyper-methylated in salivary gland adenoid cystic carcinoma [168]. Some other significant roles of *Nkx2–5* in other tumors include its interactions with *Mef2c* in ALL, with *Notch3* in T cell leukemias, dysregulated *Nkx2–5* expression in sarcomas and hypermethylation of *Nkx2–5* in breast, prostate and colon cancer [169]. It is expressed in papillary thyroid carcinoma (PTC) and reduces the expression of thyroid differentiation markers. There is age-related *Nkx2–5* methylation in normal prostate tissues and may predispose to prostate adenocarcinoma [170, 171]. In ALL, *Nkx2–5* has direct interactions with *GATA* genes and *Mef2c* oncogenic expression is influenced by *Nkx2–5*. The *Mef2c* expression inhibits apoptosis promoting *NR4A1/NUR77* expression. *Nkx2–5* is not expressed in hematopoietic stem cells, but in ALL it contributes to oncogenesis and interacts with *BCL11* [172, 173] [174]. ,Importantly, *Nkx2–5* deletions cause thyroid hypoplasia and this signifies its role in survival and proliferation as well as its various roles in different microenvironments.

#### Possible role in cardiac myxoma

Some studies have hinted towards the possible role of *Nkx2–5* in this tumor development [69]. *Nkx2.5/Csx, GATA-4, MEF2,* and *eHAND* are key involved genes in CMs. Defects in *Nkx2–5* cause abnormalities in atrial growth and development [70]. *Nkx2–5, Oct-4, Isl1, and c-kit* are upregulated and this produces cardiac progenitor stem cell-like state.

This study postulates that deviation of cardiomyocyes from cell type-specific well-differentiated state results in turning back of the cells into progenitor-like cardiac stem cells. The hallmark of this process is the upregulation of *Nkx2–5* gene expression. *Nkx2–5* has major interactions with *p53* TS gene that also prevents this tumor from becoming malignant. Cardiomyocytes have very limited proliferation potential in adult life [71]. This nature of cardiomyocytes is governed by the cell type-specific programming that also restricts the proliferative potential of this cell type after completion of cardiogenesis. *Nkx2–5* exerts vast control over proliferation. It has been found that it has wide range of functions depending on where it is expressed, as it enhances the gene expression of Mesenchymal Stem Cells (MSCs) in transplant patients and controls CPC proliferation [72].

*The heterogeneity that exists in CM may be a consequence of the multitude of roles that Nkx2–5 and other key genes/TFs and signaling pathways play in different microenvironments and in different cell types. Their dysregulations result in the deviation of cells away from cell type-specific gene expression. As the cardiac-specific combinatorial code based functioning of TFs gets dysregulated, the direction of lineages deviates from one to multiple cell types suggesting a significant role of these key genes/TFs and signaling pathways in the developmental process.*

#### Summary: unraveling the multifaceted roles of Nkx2–5

Nkx2–5, a pivotal player in cardiogenesis, orchestrates the early patterning of cardiac development, deciding atrial and ventricular fate. It controls cardiomyocyte differentiation through the JAK-STAT pathway and interacts with Mef2c to induce downstream signaling. Nkx2–5 promotes proliferation in cardiac progenitor cells (CPCs) by interacting with the Notch signaling pathway. In various tumors, Nkx2–5’s role varies, contributing to both proliferation and differentiation. This transcription factor’s presence in cardiac myxomas may relate to reprogramming cardiomyocytes into progenitor-like stem cells. Nkx2–5’s control over proliferation is vital in governing the limited proliferative potential of cardiomyocytes. Dysregulation of Nkx2–5 and other key genes/TFs and signaling pathways contributes to the heterogeneity in cardiac myxomas and their deviation from well-differentiated states, emphasizing their significance in cardiac tumor development.

### GATA4

#### Role in cardiogenesis

*GATA4* is a very important regulator of genes in the process of development. It plays a key role in the process of myocardial differentiation. The *GATA4* also plays an essential role in testicular development. The key interactions include *Nkx2–5, TBX5, SRF, HAND2, HDAC2, Erbb3, FOG-1 and FOG-2* [73, 74].

#### Proliferation-related roles

*GATA4* plays a significant role in morphogenesis and promotes cardiomyocyte survival. When *GATA4* is deleted or defective, *Erb and Erk* expression is down-regulated. They both normally play key role in EMT. *GATA4* down-regulates the *c-myc* gene expression to promote differentiation process in cardiomyocytes during development. It also regulates hypertrophic growth of heart. Although *GATA4* interacts with *p53 and p21*, it also works with *Bcl2*. This *GATA4-Bcl2* interaction promotes cardiomyocyte survival [75, 76].

#### Key interactions with tumor suppressors/ differentiation-related genes

It is expressed in both embryonic and adult cardiomyocytes. It regulates the gene expression of many downstream cardiac genes. It also maintains the cardiac function in adult heart. *GATA4* is an important regulator of terminal differentiation program in cardiomyocytes. It antagonizes *c-myc* to limit the replication potential. Multiple studies have suggested that damage to *GATA4* also damages the *Tbx5*. This damage also contributes to congenital heart defects [77]. *GATA4* plays a very significant role in differentiation process also by governing genes associated with cell-to-cell adhesion, cytoskeleton organization and extracellular matrix dynamics; this promotes them to become more differentiated and less proliferative [78]. It interacts with *p53* and *p21*, which have TS effects. It is important to note that *GATA4* interacts with CD40L and this way it is capable of inducing senescence. *GATA4* also acts as a switch to activate *NF-κB* signaling [79].

#### Contributions to combinatorial code/ cell type specific genetic-programming

*GATA4* works with other key cardiac TFs including *Nkx2–5* and *Tbx5*. *GATA4* is considered to be a key regulator of cardiac phenotype. It has upstream interactions with *BMP, FGF and Wnt* signaling pathways [175]. The significance of *GATA4* can also be estimated from the fact that when ectopically its expression is induced together with *Tbx5* and SMARCD3, this is capable of inducing genetic programming of cardiomyogenesis in non-cardiac regions of embryo. *GATA4* regulates *Mef2c* expression and acts also as *Isl1* enhancer. Note that *GATA4* which is primarily involved in cardiomyocyte differentiation interacts with *Mef2c* and *Isl1* both of which are involved in regulating progenitor and proliferation-related genes in CPCs [176]. Both *GATA4-Tbx5* and *Mef2c-Tbx5* work by triggering the gene expression of subsequent downstream cardiomyocyte-specific genes. *GATA4* and *Tbx5* are considered key regulators of cardiac gene regulatory networks. *Nkx2–5 – GATA4* complex also plays role in cardiac hypertrophy in response to stretch. This complex interaction also governs the release of Atrial and Brain Natriuretic Peptides [177].

#### Presence in other tumors

In lung cancer, it plays the role of TS, as it down-regulates the *Wnt7b* and *TGF-beta*. The presence of *SMAD4* and *GATA4* is considered to be related to poor-prognosis in esophageal adenocarcinoma. Similarly, GATA4 is also upregulated in pancreatic cancer and other cancers. Different models have shown that upregulation will increase the process of differentiation [178]. However, it fails to halt or reduce proliferation in tumor microenvironments [179, 180]. In ALL, *GATA4* has been associated with increased proliferation and inhibition of apoptosis. The predominant effect of specific genes and signaling pathways that are governing the landscape of a tumor may undermine the specific function of many differentiation-related genes [181, 182].

#### Possible role in cardiac myxoma

Primitive cardiomyocyte TFs have been detected in CM including *GATA4, Mef2c, Nkx2–5 and eHAND* [80]; they are slightly or even intensely positive in cardiac myxoma samples. In many samples, *GATA4* gene expression was dysregulated. Decline or disruptions in gene expression of key regulatory differentiation genes such as *GATA4* may have drastic impact on the overall genetic composition of differentiated cardiomyocytes. Such alterations can disrupt the delicate cell type-specific balance of expression among different types of genes/signaling pathways. This may contribute to switch the cells more towards a progenitor-like state, that is a hallmark of CM [81].

#### Summary: unraveling the multifaceted roles of GATA4

GATA4, a critical regulator in cardiogenesis, governs myocardial differentiation and is essential in testicular development. It interacts with various factors, including Nkx2–5, TBX5, SRF, HAND2, HDAC2, Erbb3, FOG-1, and FOG-2. GATA4 promotes cardiomyocyte survival, morphogenesis, and hypertrophic growth while down-regulating c-myc expression. It interacts with multiple tumor suppressors like p53 and p21. GATA4’s role extends to cardiac phenotype regulation, influencing BMP, FGF, and Wnt signaling pathways. It can induce genetic programming of cardiomyogenesis in non-cardiac regions. In tumors, GATA4 may have variable effects, acting as a tumor suppressor in lung cancer but upregulated in pancreatic cancer. In cardiac myxoma, alterations in GATA4 expression may shift cells toward a progenitor-like state, disrupting cell type-specific gene balance.

### Tbx5

#### Role in cardiogenesis

Tbx5 is one of the key regulators of cardiogenesis. It is involved in promoting differentiation of CPCs into cardiomyocytes. It interacts with *NKX2–5, GATA4 and BAF* remodeling complex. Studies in which *Tbx5* was deleted by CRISPR/Cas9 editing, showed that the cells maintained stem cell-like pluripotent state [82, 83]. *Tbx5* is a key player in switching CPCs towards developmental gene expression by inducing differentiation into cardiomyocytes. Mutations in this key TF contribute to Atrial Septal Defect (ASD). It is essential for the development of heart and limbs. It is expressed in the embryonic, adult heart and in the endocardium of left ventricle [84–86].

#### Proliferation-related roles

In the ventricle, Tbx5 expression originates from the FHF but atrial gene expression originates from *Mef2c* in the SHF. *Mef2c* plays very important role in the proliferation of CPCs [87]. *Tbx5* works with *SHH* in the formation of atrial septum. The TF *Tbx5* has a very strong relationship with *Nkx2–5,* and *Tbx5 – Nkx2–5* complex contributes to the process of cardiomyocyte differentiation. This complex also prevents activation of non-cardiac genes [88, 89].

#### Key interactions with tumor suppressors/ differentiation-related genes

Tbx5 is mutated in Holt-Oram syndrome. *Tbx5* promotes other cardiogenic TFs. It is strongly interconnected with *GATA4* and damage to *GATA4* also damages *Tbx5.* The TF *Tbx5* is so significant for the process of differentiation of cardiomyocytes that when it is defective, this contributes to the apoptosis [90, 91]. *Tbx5* interacts with *Nkx2–5, GATA4 and BAF60c* to drive expression of cardiac genes. *Tbx5* also interacts with repressor genes such as *NuRD* complex, *SALL4* and others to downregulate the expression of non-cardiac genes. Moreover, *Tbx5* induces the expression of downstream genes related to cardiomyocyte differentiation including *NPPA* and *GJA5*. Just like *Tbx5-Nkx2–5* complex, *Tbx5* also forms a complex with *GATA5* and *Mef2c* to contribute to the process of cardiomyocyte differentiation. These partnerships by Tbx5 play cell type-specific key roles in the process of development [92–94].

#### Contributions to combinatorial code/ cell type specific genetic-programming

Tbx5 works with *Nkx2–5* to promote cardiac differentiation. *Tbx5* shifts the gene expression profile more towards cardiogenesis and it also plays key role in the beating of cardiomyocytes. In the entire process of cardiac development, the gene expression of Tbx5 is maintained, whereas it also persists in the adult heart. The key interactions of Tbx5 include *Nkx2–5, GATA4, Baf60c, and Mef2c* in cardiomyocyte development. It also interacts and regulates the gene expression of a cascade of downstream genes involved in cardiac differentiation. It inhibits the gene expression of neural and other non-cardiac cell types in cardiogenesis through *Tbx5-NuRD* interaction [183].

#### Presence in other tumors

*Tbx5* inhibits cell proliferation in osteosarcoma. It is a critical regulator of oncogenesis. It has been found to suppress proliferation in Non-Small Cell Lung Cancer (NSCLC), acting as a TS. Even in normal embryonic developmental processes, its over-expression induces apoptosis and halts cell development. Tbx5 is epigenetically inhibited in colorectal cancer [184–186].

#### Possible role in cardiac myxoma

In the normal heart, the atrial expression of *Tbx5* is far greater than the ventricular and *Tbx5-Nkx2–5* forms a complex. This is very important as dysregulated expression of *Nkx2–5* is considered to play a very significant role in the development of CM. *Tbx5* forms key complexes that have a major effect in cell fate of cardiomyocytes, *Tbx5* is involved in activation and maintenance of cardiac lineage genes as well. It prevents off-target binding of TFs in cardiac development. Hence, alterations in its gene expression may have profound consequences [95, 96]. It is not expressed in CM; this may be a defining feature in CM development as *Tbx5* is one of the principal regulators of cardiomyocyte differentiation. Any dysregulation in *Tbx5* can trigger a cascade of destruction by altering the direction of cell type towards mesenchymal progenitor-like state. In the development and maintenance of cardiomyocytes, *Tbx5* suppresses the expression of genes involved in non-cardiac cell types. Hence, the dysregulations in *Tbx5* may be a major contributor in the emergence of heterogeneity in CM.

#### Summary: unraveling the multifaceted roles of Tbx5

Tbx5 is a vital regulator in cardiogenesis, inducing CPC differentiation into cardiomyocytes through interactions with Nkx2–5, GATA4, and the BAF60c complex. Mutations can lead to Atrial Septal Defect (ASD), impacting heart and limb development. Tbx5 collaborates with Mef2c in CPC proliferation and prevents activation of non-cardiac genes. It interacts with GATA4, Nkx2–5, BAF60c, and Mef2c to drive cardiac gene expression, playing essential roles in cardiomyocyte development. Tbx5, together with Nkx2–5, shifts gene expression toward cardiogenesis and is involved in cardiomyocyte beating. Dysregulated Tbx5 expression is associated with cardiac myxoma development, potentially disrupting cell fate and gene expression, contributing to heterogeneity. In tumors, Tbx5 inhibits proliferation in osteosarcoma, acts as a tumor suppressor in lung cancer, and is epigenetically inhibited in colorectal cancer.

### Mef2c

#### Role in cardiogenesis, contributions to the combinatorial code/cell type programming and key interactions

*Mef2c* works with *Nkx2–5* in controlling the differentiation of CPCs. GATA4 works also by interacting with both *Mef2c* and *Isl1*, and they both have major roles in proliferation of progenitor cells. The *Mef2c* forms complexes with both key differentiation-related genes (*GATA4 and Tbx5*) of cardiomyocytes. *Mef2c* interacts with NF-κB and downregulates its signaling in multiple cell types in endothelial cells. The role of *Mef2c* is significant because of its individual effect on proliferation and also with the complexes it forms [97, 98]. *Mef2c* contributes to activation of the TF *HAND1* [99, 100].

#### Proliferation-related roles

*Mef2c* is involved in cardiac morphogenesis, myogenesis, vascular development and neurogenesis. It contributes to maintaining differentiated state in muscle cells by working with other regulatory complexes. In hematopoiesis, *ERK* expression proportionally controls *Mef2c* expression. *Mef2c* plays oncogenic role in many cancers. One of the very important interactions of *Mef2c* includes its interactions with *Tbx5* and *GATA4*. These interactions are of immense significance as *Mef2c* also plays key role in the proliferation of CPCs. The complexes that *Mef2c* forms with *Tbx5* and *GATA4*, they contribute to switch the CPCs towards differentiated fate while sustaining the process of proliferation in cardiac development [101].

#### Presence in other tumors

Mef2c plays oncogenic role in ALL, Acute Myeloid Leukemia (AML), colon adenocarcinoma, Diffuse Large B Cell Lymphoma (DLBCL), and T-cell lymphomas. It also plays oncogenic role in prostate cancer and interacts with dysregulated notch signaling pathway. In hepatic cancer cells, it increases proliferative signaling. *Mef2c* acts as an essential transcription factor in AML oncogenesis. It interacts with *Sox2* during the process of oncogenesis in cancer stem cells [187, 188]. CDKN1B deletions frequently coincide with the expression of *Mef2c* in ALL. *Mef2c* also plays oncogenic role in Chronic Myelogenous Leukemia (CML) and imatinib abrogates its expression. Common cascade pathways (*p38 MAPKs-Mef2c*) that can result in proliferation, differentiation and apoptosis work with genes IL1R and *TGFBR* in many breast cancer subtypes. *Mef2c* and *Wnt* signaling pathway both regulate *SIX1* in Hodgkin Lymphoma. *Mef2c* exerts direct control over Socs2 [189]. The normal response of increased Mef2c expression is upregulation of *Socs2*. The *Mef2c* exerts oncogenic effects on *Socs2* in different leukemias such as AML and ALL. *Mef2c* is also upregulated in Rhabdomyosarcomas [190–192]. Another important role of *Mef2c* is also seen in pancreatic cancer. *YY1* acts as tumor suppressor, suppresses invasion and metastasis of pancreatic cancer cells by downregulating *MMP10* which is upregulated by *Mef2c*.

#### Possible role in cardiac myxoma

Multiple studies have detected *Mef2c* gene expression in CM samples. As *Mef2c* works in the form of complexes with other key regulatory genes/pathways including *GATA4, Isl1, Wnt*-pathway, its role is also governed by microenvironment. It is capable of playing oncogenic role [102]. When key differentiation-related genes such as *GATA4* become dysregulated, this may have drastic impact on the functioning of *Mef2c* which can ultimately go on to serve like an oncogene in CM landscape [103]. In such conditions, it may switch to work with *Wnt* and *Isl1* resulting in the emergence of CPC-like state that is hallmark of CM [104, 105].

#### Summary: unraveling the multifaceted roles of Mef2c

Mef2c collaborates with Nkx2–5, GATA4, Tbx5, and Isl1 in controlling CPC differentiation and proliferation during cardiogenesis. It also forms essential complexes with key differentiation-related genes. Mef2c is involved in cardiac, muscle, vascular, and neurogenesis development and has interactions with NF-κB. In cancer, Mef2c plays oncogenic roles in various types, including ALL, AML, colon adenocarcinoma, lymphomas, prostate, and hepatic cancers. It interacts with different genes and pathways in these malignancies. Mef2c expression is detected in cardiac myxoma (CM) samples, where its role may be influenced by microenvironment and the dysregulation of key differentiation genes. This could contribute to a progenitor-like state, a hallmark of CM.

### HAND1/2

#### Role in cardiogenesis, contributions to the combinatorial code/cell type programming and key interactions

*HAND1/2* is expressed in the adult heart and is downregulated in cardiomyopathies, it modulates cardiac hypertrophy and is also involved in heart, vascular, gastrointestinal tract, limb and neuronal development. *Mef2c* contributes to the activation of *HAND1. HAND1* plays a key role in neural crest development. It also interacts with *BMP4* which contributes further to the differentiation of cardiomyocytes [106, 107]. It has major interactions with *Nkx2–5* and *GATA4*. It encourages proliferation with *Nkx2–5* and when it interacts with *GATA4*, it affects differentiation of cardiomyocytes. It is important to remember that it also has a TS effect [108, 109].

#### Presence in other tumors

*HAND2* also acts as TS. It is downregulated in many tumors such as NSCLC and other cancers including ovarian, breast, gastric, colorectal, cervical, endometrial, prostate and esophageal squamous cell cancer [193]. But in the micro-environment of HCC it promotes tumor development. In the normal liver, the gene expression of *HAND2* is undetectable. But in some samples of HCC, it has been found downregulated. In HCC, *HAND2* interacts with *BMP* signaling cascade. Due to limitations of data on this role of *HAND2*, it is not possible to draw concrete conclusions about the role of *HAND2* in HCC [194]. HAND2 negatively regulates *TGFbeta, ROCK2 and JAK-STAT* pathway [195, 196].

#### Possible role in cardiac myxoma

Detected in many but not all cases of CM. It is considered to be involved in the development of CM [110]. *HAND1/2* acts as TS. Thus, it may have a possible contributing role in limiting the regenerative potential of cardiomyocytes and may have a contributing role in the benign nature of CM. This may also prevent the emergence of primary malignant tumors in cardiomyocytes.

#### Summary: unraveling the multifaceted roles of HAND1/2

HAND1/2 is expressed in the heart tissues, modulating cardiac hypertrophy. It also contributes to the vascular, gastrointestinal tract, limb, and neuronal development. It interacts with Mef2c and BMP4, promoting cardiomyocyte differentiation. HAND1/2 plays pivotal roles by interacting with Nkx2–5 and GATA4: it encourages proliferation alongside Nkx2–5 and promotes differentiation with GATA4, while also acting as a tumor suppressor (TS). In other cancers, HAND2 acts as a TS, downregulated in numerous cancer types, including NSCLC, ovarian, breast, gastric, colorectal, cervical, endometrial, prostate, and esophageal squamous cell cancer. However, in hepatocellular carcinoma (HCC), it may promote tumor development, interacting with the BMP signaling cascade. In cardiac myxoma, HAND1/2 is detected in many cases, potentially limiting cardiomyocyte regenerative potential, contributing to the benign nature of CM, and preventing primary malignant tumors in cardiomyocytes.

### MYOCD

#### Role in cardiogenesis, contributions to the combinatorial code/cell type programming and key interactions

Mostly *MYOCD* works with *p16* against the *TGF-beta* signaling, it induces growth arrest and also inhibits cellular proliferation by inhibiting *NF-κB* signaling. This is important because *MYOCD-SRF* axis forms a major complex with *Mef2c* to exert control on cardiac progenitors. This is involved in cardiomyocyte survival and maintenance of heart function. When *MYOCD* is defective, pro-apoptotic factors take over the control of cardiomyocytes. *MYOCD* is also involved in maintaining cardiac structural organization [111–113]. It interacts with *Nkx2–5* to enhance proliferation. But proliferation is downregulated when *SMAD3* gene expression is present. *MYOCD* also interacts with *NFAT, HNRNPA1, SRF and Mef2c* to enhance proliferation.

#### Role in proliferation, differentiation and in some other tumors

It inhibits stemness in NSCLC as it is an essential TS. It is downregulated in lung squamous cell carcinoma and lung adenocarcinoma. It inhibits stemness by inhibiting *TGF-beta* receptor signaling. The *SRF-MYOCD* axis is driver of well-differentiated leiomyosarcoma [197]. But *MYOCD* functions are also governed by the interactive complexes it forms with key regulatory genes. When *MYOCD* forms an interactive loop with *SMAD3/4*, it derives *TGF-beta* based Epithelial mesenchymal transition (EMT) [198, 199]. *MYOCD*, which also has TS effect, is repressed through proliferative signaling by *FOXO3A and KLF4/KLF5*. The TS *P53* also has a dose dependent regulatory repressor effect on *MYOCD*. *GSK3-beta* can inhibit *MYOCD*-dependent cardiac gene expression. The activators of *MYOCD* include *p300. MYOCD* is also inhibited by its *ERK1/2* based phosphorylation [200–203].

#### Possible role in cardiac myxoma

There are no proper data on the role of *MYOCD* in CM. But it may have a possible significant role in the process of cardiac tumorigenesis. Based on its interactions with key TFs and its role in inhibiting the stemness-related progenitor genes and signaling pathways, *MYOCD* may have a profound role in preventing the occurrence of primary tumors in cardiac tissue. As it works together with *Nkx2–5* which is expressed in CM cells, *MYOCD* may have a role in maintaining the benign nature of cardiac myxoma and in preventing the occurrence of malignant tumors in cardiac tissue.

#### Summary: unveiling the multifaceted roles of MYOCD

MYOCD primarily collaborates with p16 to counteract TGF-beta signaling, inducing growth arrest and inhibiting cellular proliferation. Through the MYOCD-SRF axis, it forms a significant complex with Mef2c, impacting the regulation of cardiac progenitors, enhancing cardiomyocyte survival, and maintaining heart function. Defects in MYOCD may lead to the dominance of pro-apoptotic factors, disrupting cardiomyocyte regulation and cardiac structural organization. Concerning proliferation, MYOCD interacts with Nkx2–5 to enhance it but downregulates when SMAD3 is present. Inhibitory interactions with NFAT, HNRNPA1, SRF, and Mef2c also contribute to proliferation. MYOCD acts as a tumor suppressor by inhibiting stemness in non-small cell lung carcinoma (NSCLC), downregulated in lung squamous cell carcinoma and lung adenocarcinoma. Although the role of MYOCD in cardiac myxoma is not well-documented, it may play a crucial part in preventing primary tumors in cardiac tissue. Its interactions with key TFs and its influence on inhibiting stemness-related genes and signaling pathways could contribute to maintaining the benign nature of cardiac myxoma and preventing malignant tumors in cardiac tissue.

### MSX2

#### Role in cardiogenesis, contributions to the combinatorial code/cell type programming and key interactions

In cardiogenesis, *MSX2* interacts with *HAND1/2* and they regulate the gene expression of each other. *MSX2* regulates survival of SHF precursors by protecting them against apoptosis. It also makes sure that there are no excessive proliferations of cardiac cells, cardiac neural crest cells and endothelial cells. It acts more as a regulator by interacting with both proliferation-related genes and differentiation related genes. *MSX1/2* are required for EMT of atrioventricular cushions and patterning of atrioventricular myocardium [114–116].

#### Role in proliferation, differentiation and in some other tumors

*MSX2* functions to maintain a balance between survival and apoptosis. Its upregulation enhances malignant phenotype [204, 205]. It also acts as transcriptional repressor. It induces EMT in pancreatic cancer [206]. *MSX2* working with *RAS* promotes cell growth. *MSX2* is downstream target of *RAS*. The *MSX2* expression is upregulated in diabetes and colorectal cancer [207, 208]. The *MSX2* interacts with *SOX2* to control cancer stem cell-like characterization in oral squamous cell carcinoma (SCC). *MSX2* represses tumor stem cell phenotypes by *SOX2* dysregulations in SCC [209]. The in vitro expression of *MSX2* has been found to inactivate *AKT* pathway to promote cell cycle arrest and apoptosis [210].

#### Possible role in cardiac myxoma

There are no proper data on the role of *MSX2* in CM. As its function is dependent on its interactions and cross-talk, it also varies with microenvironment. Hence, in CM its role is more likely to be dependent on tumor microenvironment. Such as in advanced CM, it may possibly contribute to tumorigenesis by promoting progenitor-like state.

#### Summary: unraveling the multifaceted roles of MSX2

MSX2 regulates gene expression with HAND1/2 in cardiogenesis, ensuring survival of SHF precursors and preventing excessive proliferation. It balances survival and apoptosis in proliferation, with upregulation enhancing malignancy. In diabetes and colorectal cancer, MSX2 is upregulated. MSX2 interacts with SOX2 in oral SCC to control cancer stem cell-like traits. In cardiac myxoma, MSX2’s role depends on the tumor microenvironment, potentially promoting a progenitor-like state in advanced cases.

### HOPX

#### Role in cardiogenesis, contributions to the combinatorial code/cell type programming and key interactions

*HOPX* is expressed when CPCs become committed to cardiomyocyte fate. The niche signals help regulate the committed state. It interacts with activated *SMADS* to repress *Wnt*-signaling pathway [117]. It switches the cells more towards differentiated fate of cardiomyocytes by promoting local *BMP* signals to inhibit *Wnt*-signaling pathway, thus promoting cardiomyogenesis [118, 119].

#### Role in proliferation, differentiation and in some other tumors

*HOPX* inhibits *Wnt*-signaling; this causes *HOPX* to trigger stem cell quiescence and also explains the role of *HOPX* as TS by acting as *RAS* inhibitor. The downregulation of *HOPX* expression contributes to colorectal, head, neck and other cancers. It plays a critical role in cell type homeostasis [211–213].

#### Possible role in cardiac myxoma

There are no proper data on the role of *HOPX* in CM. The dysregulations in *HOPX* may possibly serve to contribute towards CM development. The downregulation in its gene expression may alter the genetic landscape of cardiomyocytes as *HOPX* plays key roles in differentiation and also acts as a TS. *HOPX* dysregulations may lead to switching the gene expression in the direction of progenitor-like state, as it is present in CPCs.

#### Summary: unraveling the multifaceted roles of HOPX

HOPX is expressed during CPC commitment to cardiomyocytes, interacting with activated SMADS to repress Wnt signaling, promoting cardiomyogenesis. It inhibits Wnt signaling and serves as a tumor suppressor (TS) by inhibiting RAS. Downregulation of HOPX contributes to various cancers. In cardiac myxoma (CM), HOPX’s role remains unclear, but its dysregulation may influence CM development by altering the genetic landscape, potentially pushing gene expression toward a progenitor-like state present in CPCs.

## Key cardiac signaling pathways

### Wnt signaling pathway

#### Role in cardiogenesis and key interactions

*Wnt* plays a very important role in cardiac development also by contributing to planar cell polarity in cardiogenesis. The *Wnt*-signaling is also involved in adult heart remodeling. It also contributes to cardiac hypertrophy and increases ANP gene expression. Reduced *Wnt* levels have been linked to premature myocardial infarction. Wnt3a is involved in cardiac progenitor renewal. This pathway is involved in cardiogenesis and cardiac disease development [120, 121]. *Wnt*-signaling pathway promotes fibrosis in cardiac repair. This is a very important factor in defining the limitations of cardiac regeneration. The *Secreted frizzled-related protein (SFRP)* based downregulation of *Wnt/beta-catenin* is cardio-protective as it inhibits fibrosis and inflammation. This impact of *SFRP* gene expression causes EMT in post myocardial infarction state. The *Wnt/beta-catenin* pathway promotes proliferation in CPCs and its inhibition promotes differentiation [122, 123].

#### Role in proliferation, differentiation and in tumorigenesis

This pathway contributes to stemness in hematopoietic stem cells. In cancers, abnormal *Wnt*-signaling contributes to the maintenance of cancer stem cells. *Wnt/beta-catenin* is upregulated in ALL and Chronic lymphocytic leukemia (CLL). It interacts with *Notch* signaling too in cancer microenvironment. The APC TS also plays important role in regulating this signaling pathway. Inhibiting *Wnt*-pathway increases apoptosis in CLL [214–216]. In melanoma, it promotes tumor growth through abnormal *Wnt5a*. It is also upregulated in breast cancers and its upregulation silences its repressors [217, 218]. The loss of *PTEN* TS and *c-myc* amplifications are linked to abnormal *Wnt*-signaling. In tumorigenesis, this pathway derives tumor development [219].

*Wnt/beta-catenin* pathway has massive influence over other key genes such as TSs including *Numb* and it is capable of repressing the numb gene expression. This results in the maintenance of cancer stem cells. This is also one of the mechanisms for immune evasion by cancer stem cells. This pathway is also involved in EMT and is upregulated in colorectal cancer, prostate, pancreatic and many other cancers [220, 221].

#### Possible role in cardiac myxoma

When *Wnt-*signaling pathway is disrupted, it contributes to upregulation of the gene expression of progenitor-like signatures [124]. *Wnt/beta-catenin* maintains telomeres through Telomerase Reverse Transcriptase *(TERT)* gene. When this signaling pathway combines with *NF-κB* signaling pathway, it contributes to dedifferentiation into stem cell-like state [125]. As the *Wnt*-signaling also plays important role in early stages of cardiogenesis, hence this dedifferentiation-related role may have possible implications in CM development.

#### Summary: unraveling the multifaceted roles of Wnt signaling pathway

The Wnt signaling pathway is vital in cardiogenesis, influencing planar cell polarity and adult heart remodeling. It plays a role in cardiac hypertrophy, progenitor renewal, and fibrosis. Dysregulation of Wnt signaling is linked to myocardial infarction. In proliferation, it impacts stemness in hematopoietic stem cells and cancer stem cells, contributing to tumorigenesis in various cancers. The pathway interacts with Notch signaling and regulates TS genes. In the context of cardiac myxoma (CM), disrupted Wnt signaling may lead to gene expression patterns resembling progenitor-like states, potentially influencing CM development. Wnt signaling is involved in both cardiac development and tumorigenesis, making it significant in understanding CM.

### FGF signaling pathway

#### Role in cardiogenesis, proliferation and key interactions

The *FGF* Signaling Pathway is involved in the differentiation of stem cells to SHF progenitors and is also involved in the maintenance of pluripotency. These effects are based on interactions and complexes which *FGF* signaling pathway forms in order to exert effect on cell fate [126]. *FGF2* inhibits *TGF-beta1* and promotes cardio-protection. It is also involved in epicardial EMT, coronary vasculogenesis and angiogenesis through *FGF1*. The *FGF* Signaling Pathway interacts with the *IGF1/2, VEGF, BMPS, TGF-Beta, Wnt and Notch* signaling pathway. *FGF10 and FGF8* contribute to the proliferation of SHF progenitor cells [127]. The *FGF-MAPK a*xis promotes CPCs multi-potency. *FGFs* also have major interaction with *PI3K/AKT* pathway [128, 129]. In cardiogenesis, *FGF2-Wnt* complex exerts influence over human pluripotent stem cells to shift them into CPCs by suppressing *GSK3-beta* [130–132].

#### Role in differentiation and in tumorigenesis

*FGF2-BMP2* complex promotes cardiomyocyte differentiation. *Isl1-Tbx1* positively interacts with *FGF10*, which contributes to differentiation of CPCs. *Nkx2–5* negatively regulates *FGF10*, which is involved in promoting cardiomyocyte differentiation [222]. In cardiomyocyte differentiation, *GATA4* interacts with *FGF16* and suppresses proliferation potential. It also provides postnatal cardio-protection. The *FGF16* negatively regulates *FGF2-RAS-MAPK* complex [223, 224].

In postnatal adult cardiomyocytes, FGF Signaling plays very important role in modulating proliferation; *FGF1* is involved in homeostasis and remodeling [225]. FGFs have multifunctional roles ranging from proliferation, homeostasis to differentiation. FGF acts as blocker of premature CPCs differentiation. The *FGF-BMP* crosstalk plays key regulatory role in governing cardiomyocyte differentiation [226, 227]. The *FGF* Signaling Pathway is downregulated by *BMP4-MSX1* complex which promotes differentiation of neural crest cells. The *FGF* Pathway interacts with *Nkx2–5* to produce more profoundly the ventricular characteristics in the developing heart [228, 229].

#### Possible role in cardiac myxoma

The *FGF* Signaling Pathway may have significant role in CM development as loss of *FGF* causes gradual accumulation of atrial cells [133]. It is important to note that most CMs originate in the atria. The loss of *FGF* has such immense impact that it causes ectopic atrial gene expression in ventricles. One of the most important impacts of the sustained *FGF* signaling is that it acts to suppress cardiomyocyte plasticity. This may also point to the origins of CM [134].

#### Summary: unraveling the multifaceted roles of FGF signaling pathway

The FGF Signaling Pathway plays a crucial role in cardiogenesis by influencing stem cell differentiation to SHF progenitors, maintaining pluripotency, and promoting cardiomyocyte differentiation. It interacts with various signaling pathways, including IGF1/2, VEGF, BMPs, TGF-Beta, Wnt, and Notch. FGF2-Wnt complex shifts pluripotent stem cells to CPCs, suppressing GSK3-beta. FGFs, such as FGF10 and FGF8, drive SHF progenitor cell proliferation. In postnatal cardiomyocytes, FGF signaling modulates proliferation and homeostasis. FGF1 maintains adult cardiomyocyte homeostasis and remodeling. Dysregulation of the FGF pathway may contribute to CM development by promoting ectopic atrial gene expression in ventricles and suppressing cardiomyocyte plasticity. Most CMs originate in the atria, highlighting the pathway’s significance.

### BMPs

#### Role in cardiogenesis and key interactions

*BMPs* downregulate the expression of progenitor genes in CPCs. It enhances differentiation of cardiomyocyte. They induce some progenitor genes as well. The *BMP* signaling pathway downregulates *Isl1, Tbx1, FGF10* and switches the gene expression towards cardiomyocyte differentiation [135]. When *BMP*-signaling is defective, the gene expression of *HAND2 and Nkx2–5* remains unchanged [136, 137]. *BMPs i*nteract with *Nkx2–5, HAND2, Tbx2* and *Tbx20* to promote cardiomyocyte differentiation. It is also involved in epicardial EMT which is regulated by both T*GF-beta and BMPs. SMADs* negatively regulate *TGF-beta* [138, 139].

#### Role in proliferation, differentiation and in tumorigenesis

BMPs have dual role in tumorigenesis. They are capable of acting both as TSs and promotors of tumor development. This is based on microenvironment and overall profile of governing key regulatory genes. For example, the absence of *BMPs* causes the progression of colorectal carcinoma. In Barrett’s esophagus, the *BMP*-signaling pathway is upregulated. *BMP4* also contribute to neural development. *BMPs* interact with *K-RAS* and are upregulated in NSCLC [230]. *BMPs* are also involved in adult tissue homeostasis. In cardiogenesis, *BMP2* causes the differentiation of CPCs. Similarly, *BMP10* reduces the cardiomyocyte proliferation potential [231]. The gene expression of *BMP2* in cardiac cushions causes EMT myocardial patterning. The role of *BMPs* is influenced by the microenvironment [232]. The *BMP*-signaling pathway also acts on the progenitor genes. It promotes the gene expression of *Oct-4 and Nestin*. They are among the key genes involved in stem cells [233]. Another fascinating feature of *BMPs* includes their interactions with TSs such as *p53, p21, SMADs* and cause repression of *TGF-beta*. When *p53* is mutated, *Wnt*-signaling pathway is upregulated. As a result of *Wnt*-pathway upregulation, the interconnected loop of *BMP* signaling becomes dysregulated [234, 235]. *BMPs* have been found to act as TSs in Renal Cell Carcinoma, Glioblastoma, esophageal adenocarcinoma, prostate adenocarcinoma, diffuse gastric adenocarcinoma and others [236, 237].

The role of BMPs in HCC is different and of immense significance as these cells have vast regenerative potential; they contribute towards *G1 to S* transitions through cyclins [238–240].

#### Possible role in cardiac myxoma

*BMPs* may possibly have a very significant role in CM development as it is involved in cardiomyocyte differentiation during the process of cardiogenesis. It is also involved in limiting the cardiac regenerative potential. Further studies should be conducted to evaluate the role of BMPs in CM development.

#### Summary: unraveling the multifaceted roles of BMPs

BMPs are vital in cardiogenesis, promoting cardiomyocyte differentiation by downregulating progenitor genes in CPCs. They interact with Nkx2–5, HAND2, Tbx2, and Tbx20 to facilitate cardiomyocyte differentiation and regulate epicardial EMT. In tumorigenesis, BMPs exhibit a dual role, acting as both tumor suppressors and promoters based on microenvironment and gene interactions. BMPs interact with key genes like p53, p21, and SMADs to repress TGF-beta and influence cell behavior. Their role in HCC is especially significant due to their impact on G1 to S transitions. In cardiac myxoma, BMPs may play a substantial role in CM development by influencing cardiomyocyte differentiation and limiting regenerative potential, but further research is needed to fully understand this role.

### Notch signaling pathway

#### Role in cardiogenesis and key interactions

It is involved in cardiomyocyte proliferation, differentiation, cell fate specification and patterning [140]. Its specific role depends on its interactions with other key regulatory genes such as with *BMP2* to promote cardiomyocyte differentiation [141]. Similarly, it interacts with activins and *PI3K/AKT* pathway to promote mesenchymal state in CPCs. The notch pathway interacts with *p21, c-myc, snail1/2, TGF-beta* and in EMT it interacts with *Dll4, Jag1, BMP2, Alk3/6* and other key regulatory genes [142, 143]. ,In EMT, it also works through important interactions with *snail1/2-TGFbeta*. In SHF, *Notch* regulates *BMP4 and FGF8* gene expression [144–146].

#### Role in proliferation, differentiation and in tumorigenesis

The *notch* pathway in cancer contributes to the stemness of cancer stem cells [241]. It interacts with proto-oncogenes and inflammatory pathways. It also has strong cross-talk with *FGF* and *Wnt*-signaling pathways [242]. *Notch* has key interactions with many TSs such as *PTEN, P53, P21* and others. The network of these key cross-talks governs the direction of cell fate, and dysregulations in such key regulators contribute to the disease development including cancers [243, 244].

#### Possible role in cardiac myxoma

*Notch* may have possible implications in CM origins because of interactions with key cardiac TFs such as *Isl1 and Mef2c*. *Notch* expression increases postnatal cardiac survival and contributes to the proliferation of CPCs in cardiac development. The *Notch* signaling pathway governs cardiac tissue renewal by maintaining CPCs in a committed state. Further studies should be conducted to evaluate the role of Notch in CM development [1, 15, 19].

#### Summary: unraveling the multifaceted roles of notch signaling pathway

The Notch signaling pathway is crucial in cardiogenesis, influencing cardiomyocyte proliferation, differentiation, and cell fate. Its interactions with BMP2, activins, PI3K/AKT, p21, c-myc, and TGF-beta determine specific roles in CPCs and EMT. In SHF, it regulates BMP4 and FGF8 gene expression. In cancer, Notch contributes to stemness, interacting with proto-oncogenes, inflammatory pathways, FGF, and Wnt signaling, alongside multiple tumor suppressors like PTEN and P53. Dysregulations in this network impact disease development. Notch may have implications in CM due to interactions with cardiac TFs like Isl1 and Mef2c, supporting postnatal cardiac survival, CPC proliferation, and cardiac tissue renewal. Further research is needed to explore Notch’s role in CM development.

#### Occurrence of Cardiac Myxoma in Carney complex, pointing towards the significance of results in this study

The mutation in *PRKAR1A* causes myxomas and carney complex, and multiple myxomas are a feature of Carney complex [148].

*PRKAR1A* acts also as a TS. The mutated *PRKAR1A* also causes other tumors including thyroid tumors because of increase in gene expression of *RET/PTC2* signaling, multiple endocrine neoplasias and myxomas [149]. Mutations in *PRKAR1A* lead to the onset of dysregulated c-AMP protein kinase A signaling. CMs occur in 20–40% of Carney Complex patients and can occur in many chambers [147]. The nature of mutation in PRKAR1A also points towards the origin of CM as postulated in this study. This signifies how important the role of differentiation-related genes/TSs is in the maintenance of cell type-specific gene expression in cardiomyocytes. It also signifies how the defects in such key regulatory genes such as *PRKAR1A* can result in switching of cardiac cells towards a mesenchymal-like progenitor state present in CM [150, 151]. The CM development in carney complex also signifies the role of TSs and differentiation-related genes/TFs in maintenance, homeostasis of cardiomyocytes and also in tumorigenesis.

## Discussion

### Transformation of cardiomyocytes into progenitor-like state, hallmark of CM

Cardiac cells undergoing a transformation into a progenitor-like state, a distinctive hallmark of this benign cardiac tumor. This transformation is among the most significant possible ways of CM tumorigenesis. This reversion is attributed to dysregulations in key cardiac genes, transcription factors (TFs), and signaling pathways involved in the control of cardiomyocyte differentiation and maintenance of cardiac cell fate. CM develops when genes/TFs/signaling pathways with proliferation-related roles are upregulated and differentiation regulators are downregulated [7, 8]. The resulting tumor microenvironment also switches many factors towards tumorigenesis [5, 6].

The cardiac genetic landscape meticulously balances between genes, transcription factors (TFs), and pathways governing proliferation and differentiation. Dysregulations in the gene expression of key differentiation regulators, including Tbx5, GATA4, HAND1/2, MYOCD, HOPX, and BMPs, serve as the catalysts for this remarkable reprogramming. These regulatory actors orchestrate a symphony that drives the once-differentiated cardiomyocytes to abandon their mature identities and embrace a more primitive, progenitor-like phenotype [262, 263]. Dysregulation in factors like Isl1, Baf60 complex, Wnt, FGF, Notch, and Mef2c plays a pivotal role in this complex process. The Nkx2–5 and MSX2 contribute predominantly to both proliferation and differentiation of Cardiac Progenitor Cells (CPCs), are capable of serving influential roles in the landscape of CM development [264, 265]. This highlights the intricate balance between differentiation, proliferation, and regulation of cardiac cell fate. The dysregulations in the interconnected networks of genes/TFs/signaling pathways emphasizes the re-differentiation of cardiomyocytes in CM development [152, 266].

### The cross-talk and interactions in regulating the fate of cardiomyocytes

Cardiac development is a multistep developmental process. It is governed by CPCs that differentiate into cardiomyocytes. The entire process at every stage is governed by sequential unfolding of combinatorial code/ cell type-specific genetic programming which directs the cell type-specific genetic program to unfold [245]. The cascades of TFs/genes involved are governed also by cross-talk, the interaction between signaling pathways. These cross-talk based interactions cause the emergence of specific gene expression effect. The cardiac development process defines the nature and developmental architecture of cardiomyocytes [246]. This study has investigated cardiogenesis to trace the possible origins of CM and the relationship of limited cardiac regenerative potential with the rare occurrence/benign nature of tumorigenesis in cardiac tissues. The PTFs trigger the epigenetic programming in CPCs [247]. This sets in motion the unfolding of successive lineage specification. Further unfolding of the lineage, reshapes the genetic landscape towards cardiomyocyte development by establishing the commitment of cells towards differentiation. The committed CPCs emerge as a consequence of this effect. When PTFs combine with specific TFs, this causes initiation of cell program via unfolding regulatory networks. This process directs the development of CPCs towards cardiomyocytes [248].

### The governing role of proliferation, differentiation and tumor suppressor genes in regulating the cell fate

The entire process involving unfolding of progenitor genes, cell type-specific genes, and associated functioning of regulatory genes, is governed very tightly through cross-talks and interconnectedness of genes/TFs with key regulatory genes. The differentiation-related genes and TSs switch the cell circuitry more towards differentiation pathways. There exists specific combinatorial code/ genetic programming for each cell type; *Nkx2–5, Mef2c, GATA4, Tbx5* are among the key regulators of cardiomyocyte programming [249]. The combinatorial code/ cell type-specific genetic programming works also via cross-talk and tightly regulated relationships. The cross-talk of TFs with proliferation genes, differentiation/ TS genes, fate-specific genes, and others, govern the development and maintenance of cell type [250].

When the gene expression of key genes including Tbx5 that govern the programming of cell type becomes dysregulated, this can alter the homeostasis of cell type. This can lead to switching the gene expression of cardiomyocytes towards disease development such as dedifferentiation into the cardiac progenitor-like state, resulting in the emergence of CM [251].

Carney complex also signifies how differentiation/ TS genes govern cell fate and their defects can cause CM. The role of TSs in limiting cardiac regenerative and proliferative potential is also reflected by the TS-like effect exerted by the landscape of differentiation genes. The key TF *Nkx2–5*, which is a major TF in cardiogenesis works in close association with TSs by interacting with *p53* and *p21*. This interaction modulates the activity of this key TF. The *p53* is able to interact with both wild-type *Nkx2–5* and mutant *Nkx2–5* in cardiac tissue. The *p21*, which is a CDK inhibitor, works to regulate *Mef2c* expression. The cell type-specific interactions of key genes may vary in different microenvironments [252].

### Stemness and TS/ differentiation genes controlling the cell fate, and their dysregulations resulting in disease development

The stemness and differentiated state are the two extremes of the state of cell. Here we are focusing primarily on the cardiomyocytes. As cardiomyocytes are differentiated cells with very limited regenerative potential and they have to survive for very long durations in life, they must maintain themselves in the differentiated state. For that purpose, they maintain a sufficient level of gene expression of differentiation-related genes and TSs [253]. As other cell types, including neuronal cells, cardiomyocytes don’t go into proliferative phase the way skin or hepatic cells do, hence with age the cell circuitry shifts more towards the enhanced expression of differentiation-related genes and TSs. This overall exerts an increased TS-like effect. These differentiation-related genes interact with TSs and exert massive influence over other genes including those involved in proliferation. Hence, with aging there are increased degeneration-related effects in these cell types such as neurodegeneration in neurons and sigmoid shape of heart with decline in cardiac function. In order for these cell types to remain in arrested cell cycle state, the TSs exert massive influence on the genomic landscape of these cell types. But due to aging, this effect becomes more pronounced and contributes also to degenerative diseases [254].

Cell types with long survivals and limited regeneration capacity including neurons and cardiomyoctes, maintain profound gene expression of differentiation-related and TS genes. With increasing age, this predisposes these cell types towards the risk of degeneration such as Alzheimer’s disease in neurons, sigmoid shape heart with decline in cardiac function and others [255]. Due to the accumulation of dysregulations in gene expression of the key regulators that are involved in the maintenance of differentiated state, the cells switch towards stem cell-like progenitor state, that is also the hallmark of CM. This is possibly because of the same reason that an inverse relationship exists between Alzheimer’s disease and cancer in terms of molecular mechanisms and cellular pathways [256], further signifying the importance of differentiation and proliferation genes in disease development. It is already well established that TSs such as *p21, p27, p53*, and others promote and induce differentiation. The *p53* expression also induces differentiation in pluripotent stem cells by suppressing stemness transcription factors including *Oct-4, nanog* and others. Similarly, *p53 and p63* downregulate *Oct-4* and promote the process of differentiation.

The interconnectedness in the cell circuitry is so profound that mitogens/ proliferation-related genes also work through cross-talk and interconnectedness. For example, the *c-Myc* gene cooperates with *BCL2, BCR /ABL* and interacts with TSs too, including *p53* gene. Other examples include the Ras TF, as it also drives proliferation like *c-Myc*; similarly *FGFR3* works through tyrosine kinase and act as proto-oncogene. The Ras/Raf pathway and *PI3K/AKT* pathway interact with *c-Myc*, and the *c-Myc* works with *p53*. Similarly, cyclins /CDKs interact with *EGFR* resulting in massive increase in the gene expression of *Ras/Raf and PI3K/AKT/mTOR* pathway. These interactions and cross-talks also play key roles in pluripotent stem cells, development, post-natal cells, and disease development, including tumorigenesis [257].

Deviation of cardiomyocyes from cell type-specific well-differentiated state increases the risk of disease development, including the turning back of the cells into progenitor-like cardiac stem cells. Some studies have pointed to the dysregulated expression of *Nkx2–5* in tumor development. As *Nkx2–5* has major interactions with *p53*, this also prevents the tumor from becoming malignant. Such interactions have been described in detail in respective sections [258]. Cardiomyocytes have very limited proliferation potential in adult life. This nature of this cell type is governed by the cell type-specific programming that also restricts the proliferative potential of this cell type after completion of development in postnatal period [259].

### Heterogeneity in cardiac myxoma

The key genes/TFs and signaling pathways also play important roles in other cells types. Due to dysregulations and deviation in the cell type-specific genetic programing based gene expression in CM development, many key regulators of cell type go in the direction of gene expression related to the other cell types. This results in the presence of tumor heterogeneity in CM. For example, Isl1 also plays a role in other cell types. It interacts with FGFR in ganglion cells during embryogenesis, essential regulators of pancreatic morphogenesis and differentiation, and facilitates neuronal differentiation [260]. Retinoic acid promotes the *Isl1* expression in pancreatic endocrine differentiation. The *Isl1/2* defects contribute to the developmental defects in motor neurons. Shh induces Isl1 expression in neural development. Other major interactions of *Isl1 include: Ngn2, NeuroD4, NeuroM, Pax6 and Nkx2.2*. Isl1 gene expression, that is key component of CPCs, is also detected in pancreas, brain, lung, thymus, ovary [261].

The heterogeneity that exists in CM, may be because of the multitude of roles that *Nkx2–5, Isl1, GATA4, Tbx5* and other key genes/TFs and signaling pathways play in different microenvironments and in different cell types. The dysregulations in them result in the deviation of cells away from cell type-specific gene expression [262]. As the cardiac specific combinatorial code-based functioning of TFs get dysregulated, this deviates the direction of lineages from one specific to multiple cell types having significant role of these key genes/TFs and signaling pathways in their development [263].

### Interconnected landscape of key regulatory genes governing the cell type-specific roles

Increased TS/ differentiation-related gene expression limits the expression of proto-oncogenes; this results in declining the risk of tumorigenesis but simultaneously limits the regenerative capacity. The key cardiac genes such as *Nkx2–5* have major interactions with TSs. The genes/signaling pathways work in the form of association and combinations. Such combinations include the proliferating genes working with differentiation-related genes with key regulatory interactions with the TSs. This combination-based genetic functioning is very important part of cell type-specific combinatorial code/ genetic programming [264]. The heterogeneity in CM is because of the dysregulations in such key genes/TFs and signaling pathways. It is because of these disruptions in the interconnected working of key regulatory genes that disrupts the cell type-specific combinatorial functioning. This leads every key gene/TF to swing towards other cell lineage directions in which they also participate in development. The example of interconnected working is also seen in the TF *Nkx2–5*, which works in the interconnected network of *Nkx2–5 – mef2c –p53*, and includes many other interactions too. The TF *Nkx2–5* contributes to proliferation through *JAK-STAT* pathway. This *Nkx2–5* complex doesn’t work alone, it has positive feedback loop with *GATA4* and interacts with *Tbx5*. This sets in motion the unfolding of cell fate specific machinery. It also down-regulates *FGF10 and Isl1* for the promotion of differentiation. This also represses the expression of non-cardiac genes [265]. The presence of dysregulated *Nkx 2–5* is present in cancers such as ALL, because there it is working in a very different microenvironment and with different regulatory genes such as *Mef2c-Nkx2–5* complex, which plays oncogenic role. When the *Nkx2–5* combines with TSs, it promotes differentiation, cell cycle regulation, and apoptosis. Its function is dependent on microenvironment, key interactions and cross-talk with regulatory genes [152, 266].

### The targets for gene editing and epigenome editing in the development of future cardiac therapies

The decline in the gene expression of cardiac cell type-specific genes dysregulates the genetic programing of cardiac tissues involved in cell type-specific homeostasis. The multiple roles of every gene/TF begin to manifest themselves causing the emergence of heterogeneity [267, 268]. As the control exerted by the differentiating genes begin to decline, then the genetic landscape of CPCs begin to manifest. This switches the gene expression of cardiac tissues towards progenitor-like state, hallmark of the CM. The multipotent nature of CPCs begins to manifest itself in CM development. In adult cardiomyocytes, there remains a persistent expression of cardiac lineage-specific genes which maintains the cardiac tissue homeostasis [269]. This same persistent expression of the differentiation-related genes with TS effect also prevents the CM from becoming malignant. This effect possibly maintains the benign nature of primary benign cardiac tumors including CM, and overall contributes to the rare occurrence of primary cardiac tumors. The overall increased exertion of tumor suppressive-effect in this way also causes cardiomyocytes to have very limited regenerative potential because it antagonizes the expression of proliferation-related genes [270].

This study also provides the targets for gene-editing tools such as CRISPR gene editing or epigenome editing to correct or regulate the genes/signaling pathways which become dysregulated in CM development [271]. This study may serve as a map for genetic and epigenetic targets for the development of new therapeutic approaches towards reviving cardiac regenerative potential, targeting CM development and the development of cardiac organoids [272].

## Conclusions

Cardiomyocytes are one of the cell types that have very limited regenerative potential and survive for a very long duration of time. For this purpose, they continuously need to maintain themselves in a well-differentiated state. Based on the process of cardiomyocyte development in cardiogenesis, the emergence of CM is possibly governed by the dysregulations in key cardiac genes/TFs and signaling pathways. The dysregulations in differentiation related genes/TFs including *Tbx5, GATA4, MYOCD, HAND1/2, HOPX, MSX2, BMPs* have profound effect on controlling the gene expression of cardiomyocytes. They also contribute to limiting the regenerative potential of cardiomyocytes. The defective or dysregulated gene expression of such key differentiation-related genes causes the switching of cardiomyocytes towards progenitor-like state by causing upregulation of progenitor and proliferation-related genes. The key signaling pathways including *Wnt, BMPs, Notch, FGF* signaling pathways also play key regulatory roles in cardiac tissues. In cardiac development, their roles are very tightly regulated. And they work through cross-talk and interactions with cardiac-programming genes and regulators. The dysregulations in *Wnt, Notch, FGF* are capable of contributing to the process of tumorigenesis. Similarly, *BMPs* have more profound role as TSs in many cancers and are key contributors to the process of differentiation. The possible reason that cardiomyocytes are unable to easily change into primary malignant tumors, it is because they are very strongly regulated in differentiated state through a loop of multiple interconnected differentiation and TS genes. The massive influence exerted by these genes also causes the limitations in the regenerative abilities of cardiomyocytes. And many PTFs of CPCs including *Isl1, Nkx2–5* expressed in CPCs, they also function to maintain cardiac fate, or final cell type. The *Mef2c and Baf60* also don’t function independently; they are also very tightly regulated and work in the form of complexes with key TFs of cardiomyocytes. Another example of control over stemness is the presence of *MYOCD* gene expression. It exerts control over stemness-related genes and prevents them from changing into a total stem cell-like state. The benign nature and rare occurrence of CM is a possible consequence of the limited cardiac proliferative/ regenerative potential. More research is needed in this area; this can be done by developing models of cardiac organoids focused on cardiogenesis, and gene-editing them to transform into CM.

## Data Availability

All data generated or analyzed during this study are included in this published article.

## Abbreviations

BMPs: Bone Morphogenetic Proteins
CDKs: Cyclin-dependent kinases
CM: Cardiac Myxoma
CPCs: Cardiac Progenitor Cells
EGFR: Epidermal growth factor receptor
EMT: Epithelial mesenchymal transition
FHF: first heart field
FGF: fibroblast growth factor
GATA4: GATA Binding Protein 4
GSK3: Glycogen synthase kinase 3
HAND1/2: Heart and neural crest derivatives expressed protein ½
HOPX: homeodomain-only protein homeobox
ISL1: ISL LIM Homeobox 1
JAK: Janus kinase
Mef2c: myocyte enhancer factor 2C
MYOCD gene: Myocardin
Myc: MYC proto-oncogene
MSX2: Msh Homeobox 2
MAPK: Mitogen-activated protein kinase
mTOR: Mechanistic target of rapamycin
NF-κB: Nuclear factor kappa-light-chain-enhancer of activated B cells
Nkx2-5: Homeobox protein Nkx-2.5
Notch: Signaling pathway
Oct2: Octamer transcription factor 2
PI3K: Phosphoinositide-3 kinase
PRKAR1A: cAMP-dependent protein kinase type I-alpha regulatory subunit
PTEN: Phosphatase and tensin homolog
STAT: Signal transducer and activator of transcription
SHF: second heart field
Tbx5: T-box transcription factor 5
TFs: Transcription Factors
TGF-β: Transforming Growth Factor-β
TSs: Tumor Suppressors
Wnt: Signaling pathway
